# Advancing generative large language models toward discriminative performance in protein function prediction

**DOI:** 10.1186/s13059-026-04109-8

**Published:** 2026-05-21

**Authors:** Ying Lv, Yifan Xu, Gang Xu, Jianpeng Ma

**Affiliations:** 1 Shanghai AI Laboratory, Shanghai, 200030 China; 2 Multiscale Research Institute of Complex Systems, Fudan University, Shanghai, 200433 China

**Keywords:** Generative large language model, Protein language model, Protein function prediction

## Abstract

**Background:**

While generative large language models (LLMs) have revolutionized diverse research domains through their advanced semantic understanding capabilities, their applications to protein function prediction remain limited. Although significant efforts have been made to develop biological-knowledge-integrated LLMs, current approaches primarily focus on benchmarking their performances against general-purpose foundation models (e.g., ChatGPT-4o, DeepSeek-v3) rather than addressing their substantial performance gaps compared to specialized discriminative models (e.g., ESM2, ProtT5-based models).

**Results:**

We introduce OPUS-PLLM, a multitask generative LLM that establishes a sequence-to-function paradigm through natural language generation. The model integrates three components: modality encoding, modality refinement, and instruction tuning. To support its training, we construct two datasets, OPUS-InstructionCorpus and OPUS-InstructionCorpus-Evol, covering six protein functional annotations. The evaluations across five core protein function prediction tasks (spanning 18 benchmarks) demonstrate that OPUS-PLLM not only outperforms existing biological-knowledge-integrated LLMs but also surpasses specialized discriminative models in most cases.

**Conclusions:**

Our results highlight the unexplored potential of LLMs in protein function prediction and provide a robust, scalable, and generalizable solution for developing biological LLMs.

**Supplementary Information:**

The online version contains supplementary material available at 10.1186/s13059-026-04109-8.

## Background

In recent years, the rapid advancement of generative large language models (LLMs) has profoundly impacted both societal applications [1–3] and scientific research [4–7]. In protein function studies, researchers currently leverage LLMs’ semantic capabilities through two primary approaches: 1) augmenting domain-specific models (e.g., protein language models) with LLM-derived semantic information [8–11], and 2) integrating domain knowledge into LLMs themselves to create biological-knowledge-integrated LLMs [12–16]. While the former approach provides some improvements in protein representations, the latter offers more fundamental advantages through its ability to consolidate multiple biological prediction tasks within a single unified LLM. Therefore, this study focuses on the latter approach, as it provides superior scalability and generalizability.

Despite recent advances have successfully integrated biological knowledge into LLMs, the field predominantly evaluates these augmented models against general-purpose foundation models to validate the efficacy of their proposed approaches [14, 16]. Such evaluations, however, often overlook a critical limitation: although these augmented LLMs excel at generating descriptive free-text annotations of protein functions, they frequently underperform traditional discriminative models in structured, multi-classification tasks, such as subcellular localization, Gene Ontology (GO) term and Enzyme Commission (EC) number prediction. This performance gap undermines their practical utility in real-world biological applications, in which high accuracy and reliability are essential. Thus, a pivotal challenge persists: how to develop a robust training paradigm for biological-knowledge-integrated LLMs that not only leverages their innate generative capabilities but also rivals the performance of discriminative models.

In biological applications, accurately predicting protein function from sequence data remains a fundamental challenge, especially given that ~ 20% of known proteins lack functional annotations and > 40% have incomplete context-specific characterization [17]. This knowledge gap hinders many critical applications such as enzyme engineering and biomarker discovery. The exponential growth of biological data further exacerbates this challenge, necessitating advanced computational approaches to derive functional insights directly from sequence information [18, 19].

Over recent decades, protein function prediction has evolved significantly. Early approaches primarily rely on statistical and heuristic methods, some of which, for example, predict function through sequence alignment with annotated reference proteins using similarity scoring (e.g., BLAST and profile hidden Markov models (pHMMs) [20–22]). However, these traditional methods are limited by their dependence on detectable sequence homology, rendering them ineffective for proteins with few or no evolutionary relatives.

Recent breakthroughs in deep learning, particularly those in protein language models (e.g., ESM series [23, 24], ProtT5 [25], Ankh [26]), have driven a paradigm shift in protein function prediction. These models can directly infer evolutionary patterns from sequence data, achieving unprecedented prediction accuracy [27–33]. However, current approaches require training separate discriminative models for each prediction task, limiting their adaptability across diverse biological contexts. In addition, when encountering terms outside their established vocabulary, these models require retraining from scratch to incorporate the new terms into the prediction framework, which, is another limitation of discriminative models.

In this work, we present OPUS-PLLM, a multitask generative LLM for protein function prediction via a question-answering paradigm. Our approach includes three key steps: modality encoding, modality refinement, and instruction tuning. Evaluations across five core protein function prediction tasks, including subcellular localization, GO term prediction, UniProt keyword prediction, EC number prediction and functional description generation, demonstrate that OPUS-PLLM consistently outperforms both state-of-the-art biological-knowledge-integrated LLMs and specialized discriminative models across most evaluation metrics. For example, on GO term prediction task, OPUS-PLLM achieves superior performance with an average F1-score improvement of 29.90% over the next-best biological-knowledge-integrated LLM and 8.88% over top-performing discriminative models across four benchmarks. These findings signify the potential of generative LLMs and paves the way for their broader, more general applications in computational biology.

## Results

### Overview of OPUS-PLLM

In this paper, we introduce OPUS-PLLM, a multitask generative large language model (LLM) that embodies a sequence-to-function paradigm via natural language generation. The model comprises three core components: modality encoding, modality refinement, and instruction tuning. Modality encoding bridges the gap between protein sequences and functional descriptions through contrastive learning, constructing a unified cross-modal representation space. Modality refinement deepens the understanding of protein sequences by training a projection module to map sequences into the LLM’s token embedding space. Instruction tuning optimizes the generative LLM through LoRA-based fine-tuning and further refines the projection module, thereby enhancing final prediction performance.

### Performance of different models on five protein function prediction tasks

**Fig. 1:**
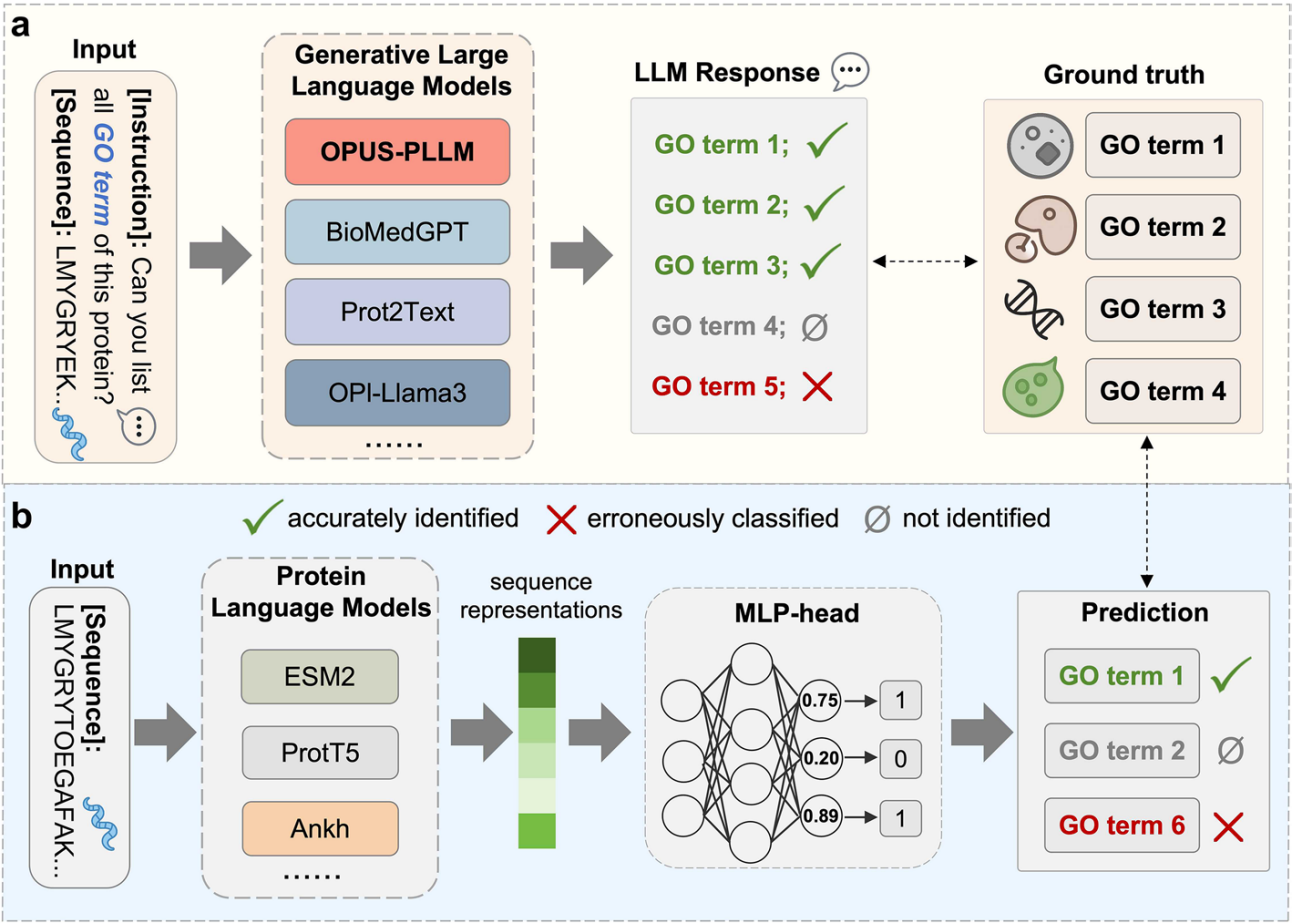
The prediction workflows of (**a**) biological-knowledge-integrated generative LLMs and (**b**) traditional specialized discriminative models based on PLM representations

In Fig. 1, we compare the prediction workflows of biological-knowledge-integrated generative LLMs (Fig. 1a) and traditional discriminative models based on protein language models (PLMs) (Fig. 1b). The former generative approach takes both protein sequence and a task-specific natural language instruction as inputs, directly generating a textual response. In contrast, the latter discriminative models first extract protein representations from a PLM, then train separate Multi-Layer Perceptron (MLP) heads for each downstream task, ultimately outputting the sigmoid value regarding the probabilities for each prediction term within a predefined vocabulary.

**Fig. 2:**
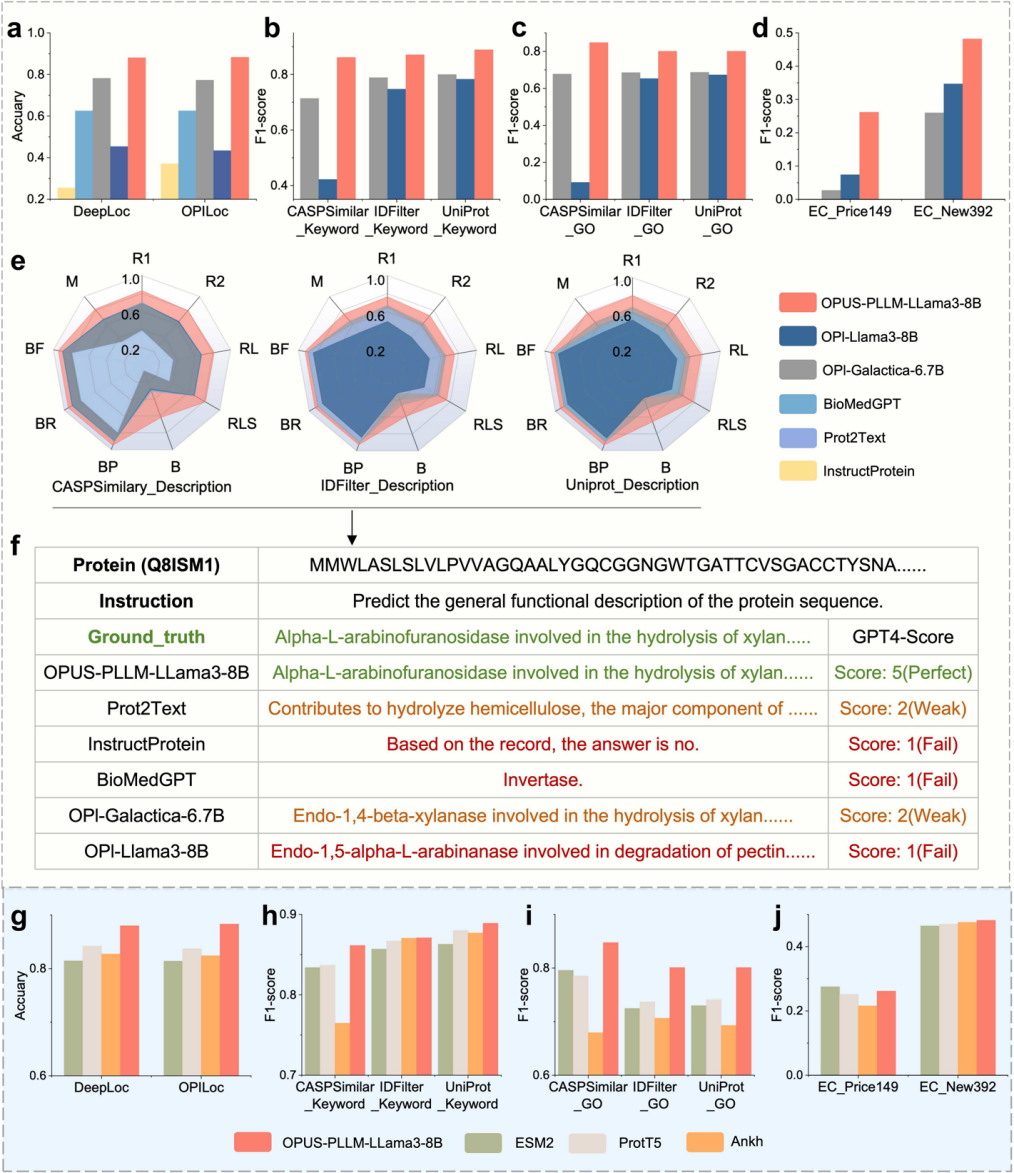
Performance comparison of different models across five protein function prediction tasks. **a**-**e** OPUS-PLLM versus state-of-the-art generative LLMs on: **a** two subcellular localization datasets, **b** three UniProt keyword datasets, **c** three GO term datasets, **d** two EC number datasets, and **e** three functional description generation datasets. **f** Some examples from functional description generation tasks. **g**-**j** OPUS-PLLM versus discriminative approaches based on different PLM representations (ESM2, ProtT5, Ankh) on: **g** two subcellular localization datasets, **h** three UniProt keyword datasets, **i** three GO term datasets, and **j** two EC number datasets

We evaluate OPUS-PLLM against five leading biological-knowledge-integrated generative LLMs (InstructProtein [14], Prot2Text [34], BioMedGPT [16], OPI-Llama, and OPI-Galactica [35]) across five critical function prediction tasks involving 13 testing datasets. Using Llama3 as the foundation model, OPUS-PLLM consistently outperforms all existing generative LLMs across all tasks (Fig. 2a-f and Additional file 1: Table S1). Notably, our model demonstrates particularly superior performance on more complex tasks, outperforming the second-best generative LLM with a relative improvement of 16.56–25.13% in Gene Ontology (GO) term prediction (Fig. 2c) and 38.90–254.61% in Enzyme Commission (EC) number prediction (Fig. 2d) in terms of F1-score. Additional file 1: Tables S2-S5 provide illustrative examples of predictions made by different generative LLMs across subcellular localization prediction, UniProt keyword prediction, GO term prediction, and EC number prediction. Additionally, in functional description generation task (Fig. 2e, f), OPUS-PLLM is also capable of delivering more accurate and biologically informative descriptions measured by multiple evaluation metrics.

We further evaluate OPUS-PLLM against traditional discriminative methods that leverage PLM representations from three state-of-the-art models: ESM2 [23], ProtT5 [25], and Ankh [26]. In contrast to our unified generative framework, these discriminative methods necessitate the development of distinct, task-specific models. Each model incorporates an MLP module derived from OPUS-GO [32] to project PLM representations onto the corresponding task labels. The results, illustrated in Fig. 2g-j and Additional file 1: Table S6, reveal that OPUS-PLLM achieves comparable or superior performance over discriminative models across various downstream tasks. These results demonstrate the effectiveness of our proposed method in bridging the gap between generative and discriminative workflows in protein function prediction. Furthermore, OPUS-PLLM’s ability to circumvent task-specific fine-tuning offers a robust, scalable, and generalizable solution for protein function prediction, making it a promising avenue for future research in this domain.

To test OPUS-PLLM’s ability to distinguish between semantically similar GO terms, we select a challenging pair: GO:0006631 (fatty acid metabolic process) and GO:0006633 (fatty acid biosynthetic process). The former term describes all biochemical reactions involving fatty acids, whereas the latter refers specifically to biosynthesis. We curate a focused test set by extracting 31 proteins from the UniProt_GO test dataset that are annotated with only one of these two terms. On this set, OPUS-PLLM correctly predicts 26 proteins and exhibits zero confusion errors between the two terms: no protein labeled with GO:0006631 is misclassified as GO:0006633, and vice versa. This result demonstrates that OPUS-PLLM can effectively separate the nuanced meanings of highly similar GO terms.

As shown in Additional file 1: Fig. S1, we conduct a detailed evaluation of OPUS-PLLM’s performance across different GO hierarchy depths. Specifically, we categorize GO terms into three groups based on their depth within the ontology: shallow (depth ≤ 2), intermediate (3 ≤ depth ≤ 6), and deep (depth ≥ 7). We then calculate the weighted average of Precision, Recall, F1-score, and Matthews Correlation Coefficient (MCC) for each depth group across all four benchmark datasets. The results show that OPUS-PLLM maintains robust predictive performance even on deep, highly specific GO terms, with no significant performance drop for terms at depth ≥ 7 compared to shallower terms.

### Performance of different models on Swiss2024-series testing datasets

**Fig. 3:**
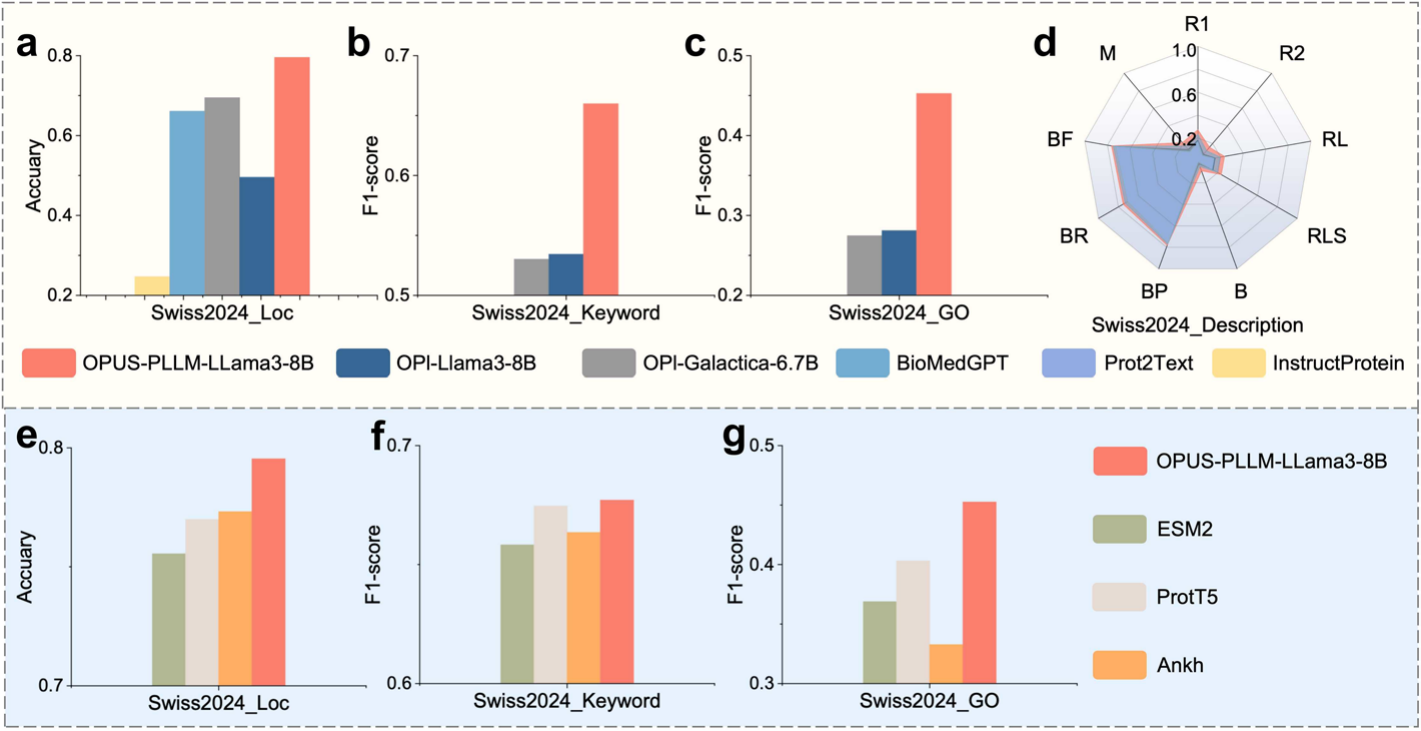
Performance comparison of different models on three Swiss2024-series testing datasets. **a**-**c** OPUS-PLLM versus state-of-the-art generative LLMs on: **a** subcellular localization prediction, **b** UniProt keyword prediction, **c** GO term prediction, and **d** functional description generation. **e–g** OPUS-PLLM versus discriminative approaches based on different PLM representations (ESM2, ProtT5, Ankh) on: **e** subcellular localization prediction, **f** UniProt keyword prediction, and **g** GO term prediction

To assess model generalizability, we evaluate OPUS-PLLM and other methods on Swiss2024-series testing datasets, containing the most recently released targets collected by this study. OPUS-PLLM exhibits a consistent superiority over all competing models across all protein function prediction tasks. Specifically, it achieves substantial improvements over the second-best generative LLMs (Fig. 3a-d and Additional file 1: Table S7), and outperforms all discriminative models across evaluated tasks (Fig. 3e-g and Additional file 1: Table S8). These findings indicate OPUS-PLLM’s robust generalization capability, establishing it as an effective tool for predicting protein functions in previously uncharacterized sequences.

### Contributions of each step in OPUS-PLLM

**Fig. 4:**
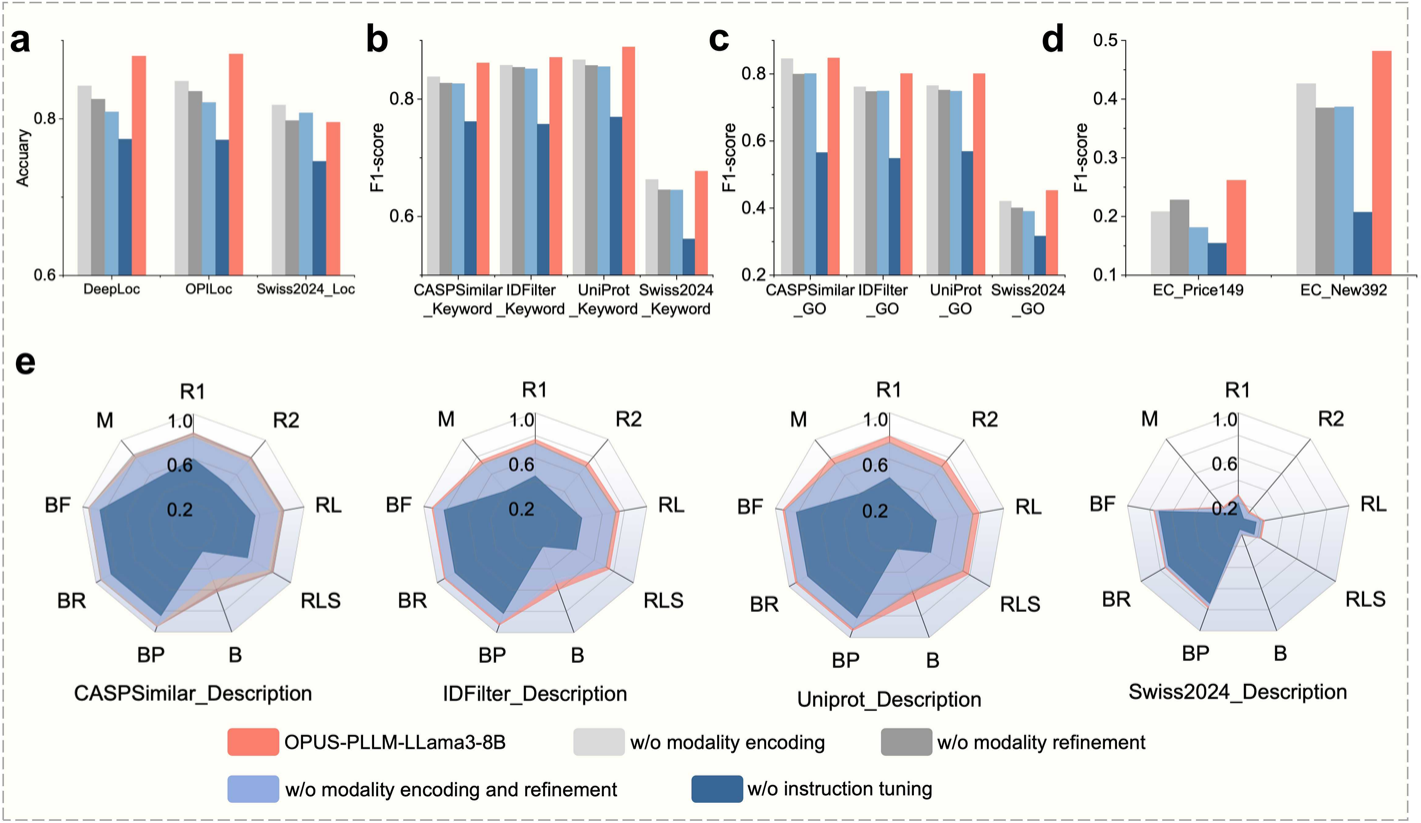
Performance comparison of OPUS-PLLM and its four ablated variants (i.e. without modality encoding, without modality refinement, without both modality encoding and refinement, and without instruction tuning) across five protein function prediction tasks. **a**-**e** OPUS-PLLM versus state-of-the-art generative LLMs on: **a** three subcellular localization datasets, **b** four UniProt keyword datasets, **c** four GO term datasets, **d** two EC number datasets, and **e** four functional description generation datasets

Through ablation studies, we evaluate the contributions of three key components in OPUS-PLLM by testing four variants: 1) without modality encoding, 2) without modality refinement, 3) without both modality encoding and refinement, and 4) without instruction tuning. As shown in Fig. 4 and Additional file 1: Table S9, instruction tuning proves the most critical for model performance. Both modality encoding and refinement individually contribute to accuracy, as their removal leads to performance degradation. In addition, ablating both simultaneously results in a more severe decline in most cases, underscoring their complementary roles in the model’s effectiveness.

Instruction tuning stage involves direct, end-to-end task adaptation, i.e., we not only optimize the projection module but also fine-tune the core LLM via LoRA to specifically follow biological instructions and generate accurate functional annotations. Consequently, it yields the most direct impact on downstream metrics. In contrast, the contributions of modality encoding and modality refinement are intentionally more foundational and preparatory. Modality encoding focuses on establishing a unified semantic alignment between protein sequences and text at the representation level. It provides a strong cross-modal initialization but does not directly train on the final task objectives. Modality refinement then adapts the projection module to better translate these aligned features into the LLM’s token space. By keeping the LLM itself frozen, this stage efficiently ensures that sequence information is injected in a consistent and structurally compatible manner. Therefore, refinement establishes a robust representational bridge, and instruction tuning specializes the entire system for high-performance, task-specific reasoning.

### Single-task vs. multi-task training strategies in OPUS-PLLM

**Fig. 5:**
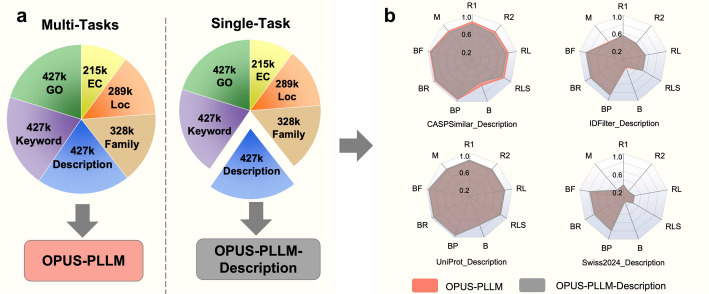
Performance comparison between single-task versus multi-task training in OPUS-PLLM. **a** Datasets used for each training strategy. **b** Performance of different models on functional description generation

To evaluate OPUS-PLLM’s performance under both single-task and multi-task learning paradigms, we compare the performance of individually trained task-specific LLMs against the unified OPUS-PLLM model trained on all tasks simultaneously (Fig. 5a). Our results show that OPUS-PLLM achieves comparable performance across all tasks compared to the specialized single-task models (Fig. 5b and Table 1). Notably, across three benchmarks, the average subcellular localization accuracy improves by 10.1% in the multi-task setting (i.e., OPUS-PLLM vs. OPUS-PLLM-Loc in Table 1). This enhancement is likely attributed to the intrinsic relationship between subcellular localization and the cellular component (CC) context within GO term prediction, which can be effectively captured through the multi-task training approach.

**Table 1:** Detailed comparison between single-task versus multi-task training strategy in OPUS-PLLM across four protein function prediction tasks

Benchmarks	Models	Accuracy	Precision	Recall	F1-Score
Subcellular Localization					
DeepLoc	OPUS-PLLM-Loc	0.771	-	-	-
	OPUS-PLLM	0.880	-	-	-
OPILoc	OPUS-PLLM-Loc	0.801	-	-	-
	OPUS-PLLM	0.883	-	-	-
Swiss2024_Loc	OPUS-PLLM-Loc	0.752	-	-	-
	OPUS-PLLM	0.795	-	-	-
Gene Ontology (GO) Term					
CASPSimilar_GO	OPUS-PLLM-GO		0.845	0.861	0.845
	OPUS-PLLM		0.846	0.863	0.848
IDFilter_GO	OPUS-PLLM-GO		0.806	0.805	0.786
	OPUS-PLLM		0.830	0.805	0.801
UniProt_GO	OPUS-PLLM-GO		0.847	0.817	0.815
	OPUS-PLLM		0.835	0.806	0.801
Swiss2024_GO	OPUS-PLLM-GO		0.477	0.498	0.454
	OPUS-PLLM		0.481	0.489	0.453
UniProt Keyword					
CASPSimilar_Keyword	OPUS-PLLM-Keyword		0.833	0.851	0.831
	OPUS-PLLM		0.884	0.859	0.862
IDFilter_Keyword	OPUS-PLLM-Keyword		0.885	0.868	0.863
	OPUS-PLLM		0.905	0.860	0.871
UniProt_Keyword	OPUS-PLLM-Keyword		0.917	0.888	0.892
	OPUS-PLLM		0.922	0.877	0.889
Swiss2024_Keyword	OPUS-PLLM-Keyword		0.692	0.705	0.674
	OPUS-PLLM		0.717	0.691	0.677
Enzyme Commission (EC) Number					
EC_Price149	OPUS-PLLM-EC		0.185	0.195	0.188
	OPUS-PLLM		0.262	0.262	0.262
EC_New392	OPUS-PLLM-EC		0.504	0.494	0.493
	OPUS-PLLM		0.495	0.479	0.482

### Evaluating representations derived from OPUS-PLLM in a discriminative manner

**Fig. 6:**
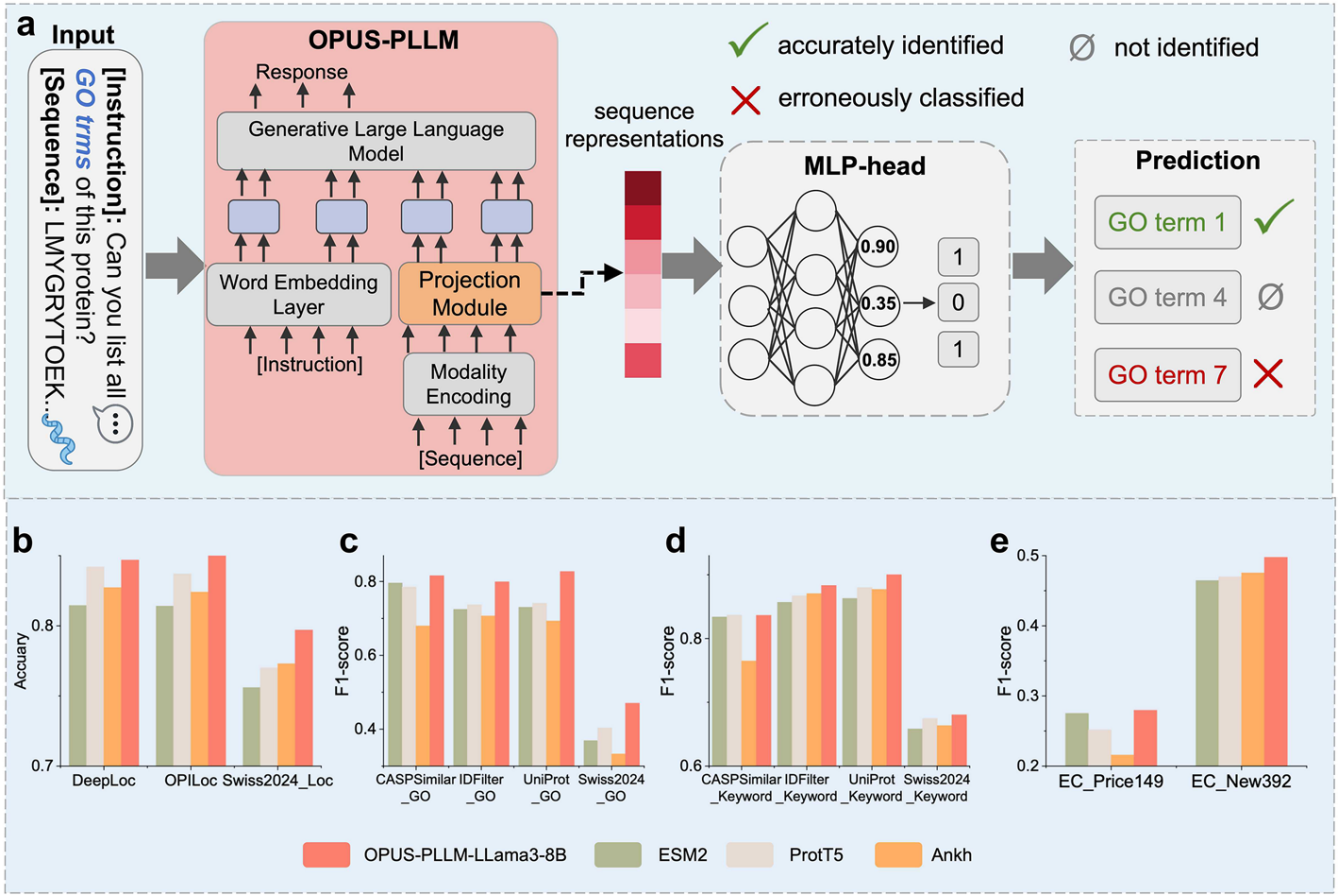
Performance comparison of discriminative models using different representations. **a** The discriminative prediction workflow utilizing OPUS-PLLM-derived representations. **b**-**e** Performance of different models on: **b** subcellular localization prediction, **c** UniProt keyword prediction, **d** GO term prediction, and **e** EC number prediction

Protein representations generated by language models serve as crucial indicators of model capability. To assess whether OPUS-PLLM improves embedding quality, we compare its representations with those from other PLMs. Following the discriminative framework described in Fig. 6a, we employ identical MLP-head (separately fine-tuned for each task) for evaluation. As shown in Fig. 6b-e and Additional file 1: Table S10, discriminative models using OPUS-PLLM representations consistently outperform PLM-based counterparts in most cases. These results confirm that OPUS-PLLM generates more biologically meaningful representations, enhancing downstream prediction performance.

### OPUS-PLLM learns the correlations between different functional annotations

To testify OPUS-PLLM’s ability to capture inter-annotation relationships, we conduct an experiment on protein GO term prediction. In addition to standard sequence and instruction inputs, we systematically provide 1–5 known GO terms as contextual prompts. Table 2 compares results between prompted (Prompt) and baseline (Base) conditions, where known annotations are counted as correct in both cases, this design isolates the model’s ability to predict remaining labels. Our results demonstrate that additional annotation context progressively improves the prediction accuracy for remaining labels, with performance gains positively correlated to the number of provided terms. These results validate OPUS-PLLM’s ability to exploit functional annotation dependencies, which is a crucial advantage of the generative paradigm.

**Table 2:** Prediction accuracy improvements for remaining GO labels when providing increasing numbers of known annotations (1–5 terms) as additional input

# of known	Models	CASPSimilar_GO	IDFilter_GO	UniProt_GO	Swiss2024_GO
0	Base	0.848	0.801	0.801	0.453
	Prompt	0.848	0.801	0.801	0.453
1	Base	0.874	0.815	0.829	0.540
	Prompt	0.892	0.830	0.847	0.573
2	Base	0.884	0.834	0.848	0.613
	Prompt	0.939	0.863	0.876	0.693
3	Base	0.895	0.852	0.862	0.660
	Prompt	0.959	0.892	0.898	0.773
4	Base	0.899	0.864	0.873	0.689
	Prompt	0.964	0.904	0.914	0.811
5	Base	0.902	0.872	0.882	0.708
	Prompt	0.969	0.916	0.925	0.836

### Performance of OPUS-PLLM using different foundation models

To evaluate the adaptability and scalability of OPUS-PLLM based on the foundation models of varying sizes, we utilize four generative LLMs with parameter scales ranging from millions to billions: DeepSeek-7B [2], Qwen3-8B-Base, and Qwen3-8B-Instruct (a reasoning-enhanced variant), Galactica-125 M, Galactica-1.3B, Galactica-6.7B [36], and Llama3-8B [1]. Across all tasks including protein subcellular localization, GO term prediction, UniProt keyword prediction, EC number prediction (Table 3), and functional description generation (Table 4), OPUS-PLLM demonstrates consistent performance when using foundation models with similar parameter counts (e.g., Galactica-6.7B and Llama3-8B), highlighting its adaptability across different generative architectures. Furthermore, the performance on Galactica models shows progressive improvement with increasing model scale, suggesting OPUS-PLLM’s potential for enhanced performance when built upon more sophisticated generative LLMs.

**Table 3:** Performance comparison of OPUS-PLLM using different foundation models (DeepSeek-7B, Qwen3-8B-Base, Qwen3-8B-Instruct (a reasoning-enhanced variant), Galactica-125 M/1.3B/6.7B, and the default Llama3-8B) on protein subcellular localization, GO term prediction, UniProt keyword prediction, and EC number prediction

Benchmarks	Models	Accuracy	Precision	Recall	F1-Score
Subcellular Localization					
DeepLoc	w/Deepseek-7B	0.843	-	-	-
	w/Qwen-8B-Base	0.883	-	-	-
	w/Qwen-8B- Instruct	0.876	-	-	-
	w/Gala125M	0.839	-	-	-
	w/Gala1.3B	0.869	-	-	-
	w/Gala6.7B	0.874	-	-	-
	w/Llama3-8B	0.880	-	-	-
OPILoc	w/Deepseek-7B	0.845	-	-	-
	w/Qwen-8B-Base	0.879	-	-	-
	w/Qwen-8B- Instruct	0.884	-	-	-
	w/Gala125M	0.842	-	-	-
	w/Gala1.3B	0.879	-	-	-
	w/Gala6.7B	0.869	-	-	-
	w/Llama3-8B	0.883	-	-	-
Swiss2024_Loc	w/Deepseek-7B	0.793	-	-	-
	w/Qwen-8B-Base	0.809	-	-	-
	w/Qwen-8B- Instruct	0.823	-	-	-
	w/Gala125M	0.811	-	-	-
	w/Gala1.3B	0.805	-	-	-
	w/Gala6.7B	0.812	-	-	-
	w/Llama3-8B	0.795	-	-	-
Gene Ontology (GO) Term					
CASPSimilar_GO	w/Deepseek-7B		0.833	0.868	0.841
	w/Qwen-8B-Base		0.851	0.861	0.850
	w/Qwen-8B- Instruct		0.840	0.861	0.844
	w/Gala125M		0.815	0.834	0.820
	w/Gala1.3B		0.827	0.862	0.836
	w/Gala6.7B		0.832	0.863	0.838
	w/Llama3-8B		0.846	0.863	0.848
IDFilter_GO	w/Deepseek-7B		0.819	0.815	0.798
	w/Qwen-8B-Base		0.844	0.822	0.817
	w/Qwen-8B- Instruct		0.840	0.821	0.814
	w/Gala125M		0.769	0.755	0.743
	w/Gala1.3B		0.827	0.796	0.794
	w/Gala6.7B		0.791	0.799	0.777
	w/Llama3-8B		0.830	0.805	0.801
UniProt_GO	w/Deepseek-7B		0.823	0.815	0.800
	w/Qwen-8B-Base		0.852	0.831	0.824
	w/Qwen-8B- Instruct		0.848	0.831	0.821
	w/Gala125M		0.771	0.753	0.743
	w/Gala1.3B		0.824	0.797	0.792
	w/Gala6.7B		0.828	0.806	0.799
	w/Llama3-8B		0.835	0.806	0.801
Swiss2024_GO	w/Deepseek-7B		0.471	0.499	0.451
	w/Qwen-8B-Base		0.487	0.509	0.464
	w/Qwen-8B- Instruct		0.489	0.508	0.463
	w/Gala125M		0.456	0.417	0.405
	w/Gala1.3B		0.476	0.490	0.449
	w/Gala6.7B		0.472	0.489	0.445
	w/Llama3-8B		0.481	0.489	0.453
UniProt Keyword					
CASPSimilar_Keyword	w/Deepseek-7B		0.867	0.831	0.837
	w/Qwen-8B-Base		0.796	0.876	0.825
	w/Qwen-8B- Instruct		0.821	0.873	0.835
	w/Gala125M		0.819	0.837	0.816
	w/Gala1.3B		0.888	0.853	0.863
	w/Gala6.7B		0.879	0.837	0.848
	w/Llama3-8B		0.884	0.859	0.862
IDFilter_Keyword	w/Deepseek-7B		0.887	0.862	0.862
	w/Qwen-8B-Base		0.903	0.877	0.878
	w/Qwen-8B- Instruct		0.905	0.879	0.879
	w/Gala125M		0.862	0.874	0.853
	w/Gala1.3B		0.881	0.871	0.863
	w/Gala6.7B		0.890	0.878	0.871
	w/Llama3-8B		0.905	0.860	0.871
UniProt_Keyword	w/Deepseek-7B		0.909	0.885	0.886
	w/Qwen-8B-Base		0.920	0.898	0.899
	w/Qwen-8B- Instruct		0.922	0.899	0.901
	w/Gala125M		0.890	0.854	0.859
	w/Gala1.3B		0.909	0.877	0.882
	w/Gala6.7B		0.913	0.877	0.884
	w/Llama3-8B		0.922	0.877	0.889
Swiss2024_Keyword	w/Deepseek-7B		0.689	0.693	0.665
	w/Qwen-8B-Base		0.703	0.705	0.680
	w/Qwen-8B- Instruct		0.694	0.699	0.672
	w/Gala125M		0.664	0.690	0.650
	w/Gala1.3B		0.689	0.704	0.670
	w/Gala6.7B		0.681	0.698	0.663
	w/Llama3-8B		0.717	0.691	0.677
Enzyme Commission (EC) Number					
EC_Price149	w/Deepseek-7B		0.342	0.342	0.342
	w/Qwen-8B-Base		0.262	0.262	0.262
	w/Qwen-8B- Instruct		0.292	0.295	0.293
	w/Gala125M		0.188	0.188	0.188
	w/Gala1.3B		0.255	0.255	0.255
	w/Gala6.7B		0.349	0.346	0.347
	w/Llama3-8B		0.262	0.262	0.262
EC_New392	w/Deepseek-7B		0.537	0.517	0.523
	w/Qwen-8B-Base		0.504	0.484	0.486
	w/Qwen-8B- Instruct		0.529	0.514	0.514
	w/Gala125M		0.315	0.328	0.311
	w/Gala1.3B		0.476	0.471	0.468
	w/Gala6.7B		0.545	0.533	0.536
	w/Llama3-8B		0.495	0.479	0.482

**Table 4:** Performance comparison of OPUS-PLLM using different foundation models (DeepSeek-7B, Qwen3-8B-Base, Qwen3-8B-Instruct (a reasoning-enhanced variant), Galactica-125 M/1.3B/6.7B, and the default Llama3-8B) on functional description generation

Benchmarks	Models	R1	R2	RL	RLS	B	BP	BR	BF	M
Functional Description										
CASPSimilar_Description	w/Deepseek-7B	0.814	0.766	0.789	0.794	0.566	0.943	0.941	0.942	0.795
	w/Qwen-8B-Base	0.802	0.755	0.778	0.779	0.523	0.937	0.943	0.939	0.794
	w/Qwen-8B- Instruct	0.841	0.810	0.828	0.828	0.622	0.951	0.954	0.952	0.838
	w/Gala125M	0.744	0.695	0.725	0.724	0.339	0.931	0.915	0.922	0.711
	w/Gala1.3B	0.822	0.786	0.808	0.807	0.610	0.944	0.948	0.946	0.810
	w/Gala6.7B	0.824	0.795	0.813	0.812	0.629	0.947	0.949	0.948	0.818
	w/Llama3-8B	0.832	0.795	0.817	0.816	0.618	0.947	0.948	0.947	0.816
IDFilter_Description	w/Deepseek-7B	0.767	0.730	0.757	0.757	0.610	0.932	0.926	0.929	0.750
	w/Qwen-8B-Base	0.779	0.745	0.770	0.769	0.534	0.928	0.931	0.928	0.767
	w/Qwen-8B- Instruct	0.785	0.749	0.773	0.773	0.628	0.937	0.933	0.934	0.769
	w/Gala125M	0.716	0.669	0.704	0.704	0.510	0.919	0.908	0.912	0.686
	w/Gala1.3B	0.747	0.711	0.738	0.739	0.573	0.927	0.919	0.923	0.728
	w/Gala6.7B	0.767	0.728	0.757	0.757	0.577	0.933	0.925	0.929	0.750
	w/Llama3-8B	0.766	0.729	0.756	0.756	0.596	0.928	0.925	0.926	0.752
UniProt_Description	w/Deepseek-7B	0.806	0.773	0.797	0.797	0.690	0.942	0.937	0.939	0.792
	w/Qwen-8B-Base	0.824	0.792	0.814	0.814	0.669	0.943	0.943	0.942	0.815
	w/Qwen-8B- Instruct	0.821	0.790	0.811	0.812	0.664	0.942	0.944	0.942	0.814
	w/Gala125M	0.707	0.653	0.693	0.693	0.508	0.917	0.903	0.909	0.679
	w/Gala1.3B	0.765	0.725	0.755	0.755	0.587	0.932	0.923	0.927	0.744
	w/Gala6.7B	0.785	0.749	0.775	0.775	0.623	0.938	0.930	0.933	0.767
	w/Llama3-8B	0.796	0.760	0.785	0.785	0.656	0.935	0.935	0.934	0.784
Swiss2024_Description	w/Deepseek-7B	0.268	0.158	0.234	0.234	0.094	0.787	0.746	0.764	0.202
	w/Qwen-8B-Base	0.284	0.168	0.244	0.245	0.120	0.776	0.754	0.762	0.225
	w/Qwen-8B- Instruct	0.279	0.163	0.240	0.240	0.113	0.778	0.752	0.762	0.218
	w/Gala125M	0.238	0.132	0.209	0.209	0.045	0.784	0.735	0.757	0.167
	w/Gala1.3B	0.262	0.151	0.228	0.228	0.067	0.790	0.746	0.766	0.189
	w/Gala6.7B	0.264	0.153	0.231	0.231	0.073	0.788	0.746	0.765	0.194
	w/Llama3-8B	0.273	0.157	0.236	0.235	0.090	0.781	0.751	0.764	0.210

### Performance of OPUS-PLLM against leading general-purpose large language models

As detailed in Additional file 1: Tables S11-S12, we have conducted a series of extended benchmark comparisons. These evaluations include recent, high-performing open-source models as well as general-purpose large language models enhanced with retrieval-augmented generation (RAG). For the RAG-based evaluations, the training dataset of OPUS-PLLM served as the search corpus.

Our comparative analysis demonstrates that OPUS-PLLM achieves superior performance over advanced foundation models and their RAG-augmented versions in all evaluated cases. This consistent lead highlights a core challenge in the field: general-purpose language models, even with explicit reasoning modules, are fundamentally limited in their ability to derive biochemical meaning from sequence data, which they process merely as arbitrary token strings. The primary objective of our approach is to bridge this semantic and functional divide.

### Performance of OPUS-PLLM-Evol on multiple-choice question answering (MCQA) task

To eliminate subjective scoring and enhance evaluation reproducibility, the multiple-choice question answering (MCQA) paradigm has been adopted in general-domain LLM evaluation (e.g., MMLU [37], MMLU_PRO [38], GPQA [39]). This approach enables precise quantification of problem-solving capabilities by presenting models with fixed answer choices, thereby restricting response generation to predefined options and minimizing variability. These features collectively position MCQA as the current gold standard for objective LLM evaluation.

**Fig. 7:**
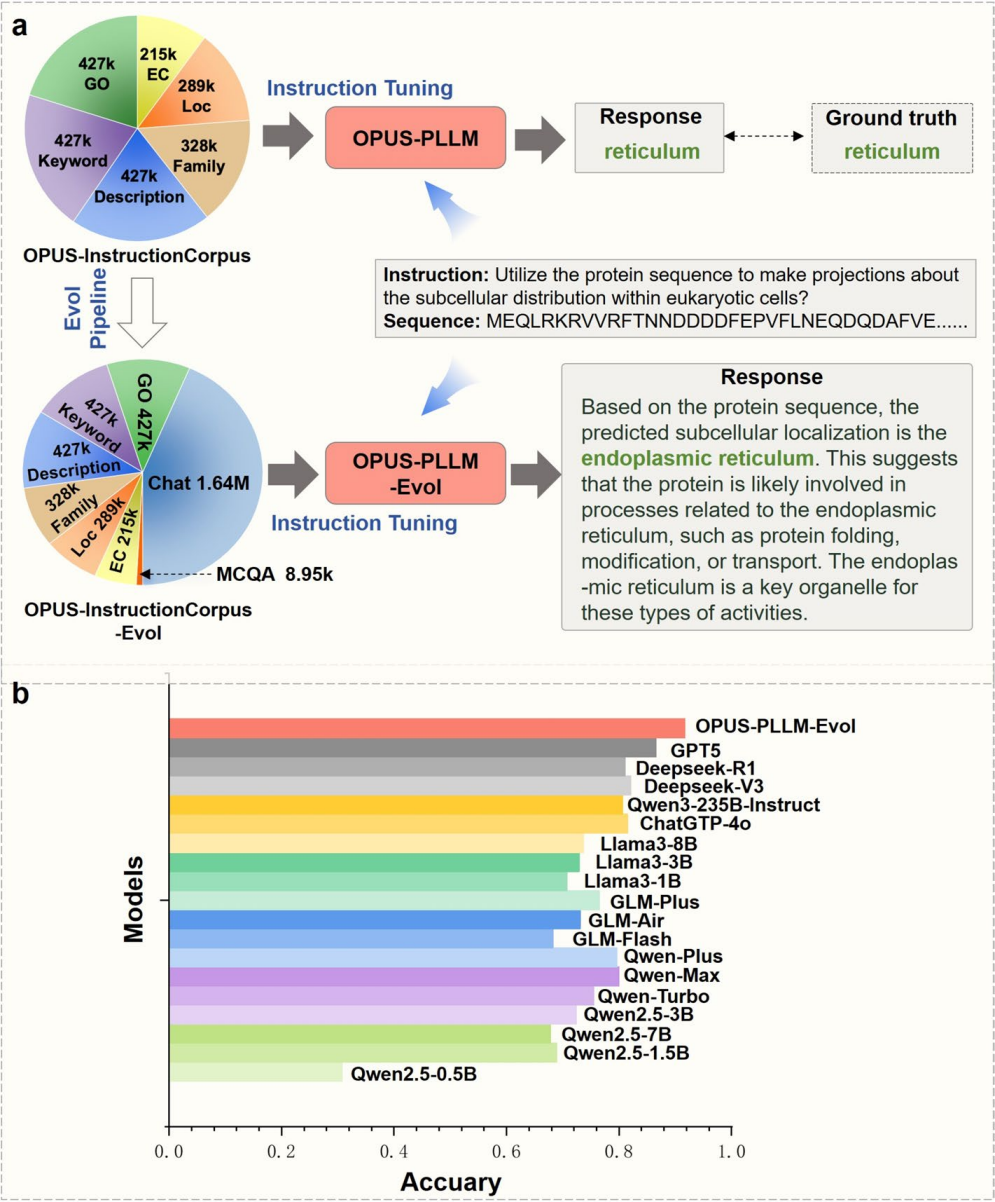
Overview of multiple-choice question answering (MCQA) task. **a** The OPUS-InstructionCorpus-Evol dataset used in instruction tuning step of OPUS-PLLM-Evol. **b** Comparative MCQA accuracy across state-of-the-art generative LLMs

To validate our framework’s training paradigm for developing generalizable biological LLMs, we introduce OPUS-InstructionCorpus-Evol (Fig. 7a), a large-scale dataset comprising 3.75 M instruction-sequence-response triples generated with the assistance of GLM4-Plus [40]. Expanding upon the original OPUS-InstructionCorpus (focused on predefined tasks), this dataset additionally incorporates 1.64 M diverse “Chat” examples, produced via simulated interactions between a domain-expert bot (with biological reasoning) and a human user (posing real-world queries). The interactions are driven by randomly sampled annotations to ensure diversity, while leveraging the GLM4-Plus language model to dynamically simulate nuanced, context-aware personas for both participants. Meanwhile, we incorporate some MCQA examples, each containing a protein sequence, a contextually grounded instruction, and four options (one correct, three plausible distractors). To ensure dataset quality, we implement a two-stage filtering mechanism combining rule-based checking and LLM-assisted fact-checking: first, a rule-based filtering step automatically removes samples that are structurally invalid, have anomalous formats, or conflict with known annotation fields. Second, we employ a specially designed fact-checking prompt to leverage the LLM for consistency verification between the generated content and the original database annotations/established biological knowledge. Samples with contradictions or inconsistencies are further filtered out. Output-first approach [41] is employed to generate each MCQA triplet. This enhanced variability equips models to handle flexible biological applications beyond narrow predefined tasks.

For evaluation, we introduce Swiss2024-MCQA, a MCQA benchmark derived from the Swiss2024-series testing datasets using GLM4-Plus, comprising 1,684 rigorously validated biological questions. Each question features four validated response options (one correct and three distractors), providing a robust evaluation framework for biological reasoning tasks.

We develop OPUS-PLLM-Evol through instruction tuning on the OPUS-InstructionCorpus-Evol dataset, building upon the base model established in the initial two training phases of OPUS-PPLM. Comparative results in Fig. 7b reveal that OPUS-PLLM-Evol achieves superior performance to general-purpose foundation models. Note that, the accuracy of aforementioned biological-knowledge-integrated LLMs on this MCQA task are almost zero, as these models are designed exclusively for predefined tasks and lack the capability to handle open-ended biological questions. Therefore, the results demonstrate that our training paradigm is capable of effectively producing models with robust protein sequence comprehension, instruction-following capability, and biological reasoning skills.

## Discussion

While generative large language models (LLMs) have revolutionized numerous fields through their semantic understanding capabilities, their applications to protein function prediction have remained limited. Current biological-knowledge-integrated LLMs still considerably lag behind traditional discriminative models, hindering their practical applications.

To address this, we present OPUS-PLLM, which contains three major components: modality encoding, modality refinement, and instruction tuning. By testing on 17 benchmarks, the results show that OPUS-PLLM achieves state-of-the-art performance on five critical protein function tasks (i.e. subcellular localization, UniProt keyword prediction, GO term prediction, EC number prediction, and functional description generation), substantially better than other biological-knowledge-integrated LLMs (Figs. 2a, 3a-d). Notably, in most cases, OPUS-PLLM also outperforms discriminative approaches utilizing protein language model (PLM) representations from ESM2, ProtT5, and Ankh (Figs. 2g, 3e-g), effectively bridging the performance gap between generative and discriminative approaches.

We also test OPUS-PLLM under both single-task and multi-task learning paradigms. The results show comparable performance across both settings, with the multi-task model exhibiting potential benefits from semantic relationships between related tasks (Fig. 5 and Table 1). These findings highlight the advantage of leveraging multi-task learning for protein function prediction and suggest that incorporating additional functionally relevant data could further enhance generative LLM performance. Furthermore, we also find that OPUS-PLLM may effectively leverage functional annotation dependencies (Table 2), which is a key advantage inherent to the generative paradigm.

In addition, our results further emphasize two key advantages of OPUS-PLLM: 1) good adaptability and scalability across foundation models of varying scales (Tables 3 and 4), and 2) strong generalization capability, as evidenced by its superior performance on multiple-choice question answering (MCQA) task (Fig. 7). These advantages collectively validate the training paradigm in this work for developing generalizable biological LLMs with robust protein sequence understanding, precise instruction-following, and reliable reasoning abilities.

The primary goal of this work is not to “defeat” highly optimized discriminative models, which have been refined over years to incorporate domain-specific knowledge such as structural information [42] and advanced feature extraction [32, 43]. Instead, we aim to substantially narrow the performance gap between generative and discriminative paradigms, demonstrating that LLMs can achieve competitive results on challenging multi-label protein function prediction tasks. Our comparative analysis with leading discriminative methods [28, 32, 42–49] shows that, in most cases, OPUS-PLLM achieves performance that is comparable to or superior to that of these specialized architectures (Additional file 1: Table S13 and Additional file 1: Table S14). These results underscore the significant potential of generative models in this field.

As supported by existing literature, LLMs excel at extracting rich semantic information from the textual annotations of proteins. This enhances the quality of protein representations and provides a strong foundation for prediction. However, general-purpose LLMs often underperform on domain-specific downstream tasks and inherently lack deep protein-related knowledge (Additional file 1: Table S11 and Additional file 1: Table S12). Consequently, specialized fine-tuning serves as the pivotal and enabling step for their application. Therefore, their primary advantage over discriminative models is not inherent superiority but their potential, when effectively fine-tuned to integrate biological knowledge, to bridge powerful semantic understanding with the precision required for accurate functional annotation, a paradigm that discriminative models do not natively support.

To further assess performance on rare (long-tail) labels, we employ the Matthews Correlation Coefficient (MCC), a robust metric for imbalanced classification. As shown in Additional file 1: Table S15, our method outperforms its sequence-based discriminative baseline, which also uses ESM2-derived features, in both mean MCC and low-frequency-term MCC. This highlights the strength of generative models over discriminative ones for predicting rare functional labels under same input settings.

Our results show no substantial performance difference between Qwen3-8B-Instruct and other models (Tables 3 and 4). However, we argue this does not fully reflect the potential of reasoning-augmented architectures. A key contributing factor is the absence of explicit Chain-of-Thought (CoT) supervision in our current training data, which limits the model’s ability to leverage structured reasoning for protein function prediction tasks. In future work, we plan to construct and integrate targeted CoT training data, which will enable more effective utilization of reasoning-based foundation models within our framework and facilitate a more meaningful evaluation of their performance gains. Furthermore, we will incorporate structured biological knowledge graphs (e.g., GO term hierarchies) to explicitly guide models through logical, knowledge-augmented inference when encountering novel terms. This enhancement aims to unlock the generative paradigm’s inherent ability to compose meaning from structured relationships, thereby enabling robust extrapolation beyond existing vocabularies.

In OPUS-PLLM, protein-level representations are derived by averaging residue-level embeddings. While this captures overall sequence characteristics and supports robust function prediction, it inherently smooths out localized signals. Consequently, the current model is not optimized to detect subtle functional changes caused by single-point mutations. Addressing this would require more fine-grained strategies, such as preserving residue-level features or incorporating localized attention mechanisms, but would increase computational cost and require specialized mutation datasets. This represents a valuable direction for future work, especially for applications requiring residue-specific functional analysis.

Drawing from our findings, we outline two promising avenues for future investigation. First, the consistent scaling of performance observed across the size of foundation models (125 M to 8B parameters) strongly suggests that deploying OPUS-PLLM on larger architectures (e.g., Llama3-70B or future billion-scale LLMs) could significantly elevate performance of this field. Second, although OPUS-PLLM currently relies primarily on linear sequence data, which is inherently informative, there is a possible potential for enhancing its predictive accuracy by integrating additional modalities, particularly high-quality structural information, if available. This multimodal approach could lead to more accurate protein function predictions, leveraging the complementary strengths of different data types.

## Conclusions

In conclusion, OPUS-PLLM successfully establishes a new generative paradigm for protein function prediction, achieving performance parity with state-of-the-art discriminative methods while offering superior generalizability. Our findings not only demonstrate the transformative potential of generative LLMs in computational biology, but also provide a foundation for developing unified, task-agnostic models that can advance our understanding of protein function at scale.

## Methods

**Fig. 8:**
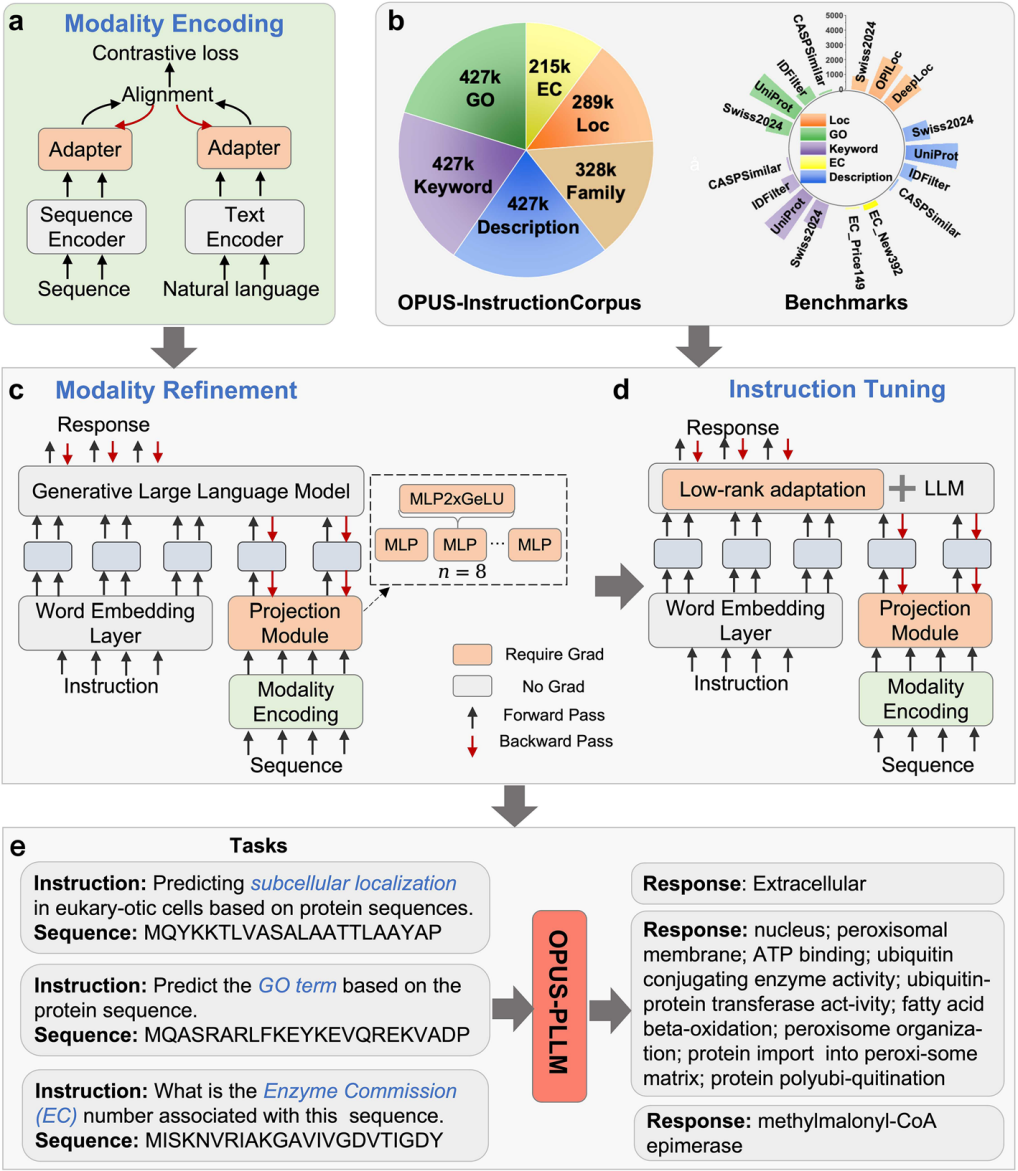
The framework of OPUS-PLLM for protein function prediction. **a** Modality encoding establishes a shared representation space between protein sequences and natural language through contrastive learning with modality-specific adapters. **b** Training resources in OPUS-InstructionCorpus for next two steps, including 2.11 million instruction pairs spanning six biological topics (GO term, UniProt keyword, functional description, protein family, subcellular localization, and EC number), and 17 benchmark datasets for evaluation. **c** Modality refinement projects protein representations into a frozen LLM’s token space via a trainable projection module, creating unified sequence-text representations. **d** Instruction tuning employs LoRA to efficiently adapt the generative LLM while further optimizing the projection module using instruction-sequence-response triplets. **e** Examples of OPUS-PLLM

OPUS-PLLM includes three key steps (Fig. 8): 1) Modality encoding bridges the protein sequence-functional description gap through contrastive learning on sequence-text pairs, creating a unified cross-modal representation space (Fig. 8a). 2) Modality refinement enhances protein sequence understanding by processing 2.11 million instruction samples from OPUS-InstructionCorpus (Fig. 8b) via a projection module that maps sequences into the LLM’s token embedding space (Fig. 8c). 3) Instruction tuning optimizes generative LLM (default: Llama3-8B [1]) using LoRA-based adaptation and refines the projection module to boost final prediction quality (Fig. 8d).

### Modality encoding

The modality encoding step establishes a unified representation space for protein sequences and textual descriptions, enabling cross-modal semantic alignment. The dataset consists of paired protein sequences and functional descriptions. The dataset used in this step (see Dataset section for more details) consists of paired protein sequences and functional descriptions, represented as $P = \left(S, T\right)$, where $S = \left\{{s}_{1}, {s}_{2}, \dots ,{s}_{n}\right\}$ is a set of protein sequences, $T = \{{t}_{1}, {t}_{2}, \dots ,{t}_{n}\}$ is the corresponding set of textual descriptions.

In this step, we use ESM2 [23], a state-of-the-art protein language model (PLM), to encode protein sequences into 1280-dimensional representations,

$${Emb}_{seq}^{ESM}=ESM2\left({X}_{seq}\right)=\frac{1}{n}\sum_{i=1}^{n}{ESM2\left({X}_{seq}\right)}_{i}$$

where ${X}_{\mathrm{s}\mathrm{e}\mathrm{q}}$ denotes the input protein sequence, and $n$ is the number of residues in it.

Meanwhile, we employ Llama3 [1], a leading open-source generative LLM, to extract semantic representations of textual descriptions,

$${Emb}_{text}^{Llama}= mean({Llama}_{first}({X}_{text})+ {Llama}_{last}({X}_{text}))$$

where ${X}_{\mathrm{t}\mathrm{e}\mathrm{x}\mathrm{t}}$ is the input text description, ${Llama}_{first}({X}_{text})$ and ${Llama}_{last}({X}_{text})$ represent the first and last layer representations of the Llama3, respectively.

Then, we employ a linear projection layer as the modality adapter, following established practices that have demonstrated both effectiveness and computational efficiency [10].

$${Z}_{seq }= {W}_{seq}{ Emb}_{seq}^{ESM }+ {b}_{seq}$$

$${Z}_{text }= {W}_{text}{Emb}_{text}^{Llama }+ {b}_{text}$$

where ${W}_{\mathrm{s}\mathrm{e}\mathrm{q}}$ and ${W}_{\mathrm{t}\mathrm{e}\mathrm{x}\mathrm{t}}$ are projection matrices, and ${b}_{\mathrm{s}\mathrm{e}\mathrm{q}}$ and ${b}_{\mathrm{t}\mathrm{e}\mathrm{x}\mathrm{t}}$ are bias terms.

To achieve semantic alignment between protein sequences and their textual descriptions, we utilize a contrastive learning framework [50]. Contrastive learning facilitates semantic coherence by optimizing pairwise distances within a shared embedding space: it minimizes the semantic disparity between positive pairs (e.g., protein sequence and its corresponding textual description), while simultaneously maximizing the divergence between negative pairs, which consist of mismatched protein-text combinations. Through this optimization, the model learns to project semantically related proteins and texts to nearby regions in the shared representation space, while pushing unrelated pairs apart. Despite the inherent modality differences between sequences and language, this framework enables them to occupy similar positions in the latent space when they convey equivalent biological meaning.

The InfoNCE loss function is adopted in contrastive learning, which is defined as:

$$L = -\frac{1}{N} \sum \mathit{log}\frac{\mathit{exp}\left({Z}_{seq} \cdot { Z}_{text+} / \tau \right)}{{\sum}_{i=0}^{k}\mathit{exp}\left({Z}_{seq} \cdot { Z}_{text-}^{i } / \tau \right) }$$

where $\tau$ is a temperature parameter controlling similarity sensitivity.

### Modality refinement

The modality refinement step further enhances the integration of protein sequence features with linguistic context to better capture biological knowledge. The process utilizes the OPUS-InstructionCorpus dataset (see Dataset section for more details) containing 2.11 M triplets of the form $\left({X}_{instruction},{X}_{seq},{X}_{response}\right)$, where ${X}_{instruction}$ refers to task-specific natural language query about the protein, ${X}_{seq}$ is the input protein sequence, and ${X}_{response}$ is the target functional annotations.

For an input protein sequence ${X}_{seq}$, we first extract features using the frozen modality encoder ${Z}_{seq}={f}_{encoder}({X}_{seq})$. Then, a trainable multi-head Multi-Layer Perceptron (MLP) module derived from MLP2xGeLU [51, 52] projects ${Z}_{seq}$ into $n=8$ generative-LLM-compatible tokens, each MLP module comprises two linear layers with an intermediate GeLU activation.

$${H}_{seq} = \left[{H}_{seq}^{1},\dots ,{H}_{seq}^{j},\dots ,{H}_{seq}^{n}\right]$$

$${H}_{seq}^{j}={W}_{2}^{j}\left(GeLu\left({W}_{1}^{j}\cdot {Z}_{seq}\right)+{b}_{1}^{j}\right)+{b}_{2}^{j}$$

$${W}_{proj}^{j}=\{{{W}_{1}^{j},W}_{2}^{j}, {b}_{1}^{j},{b}_{2}^{j}\}$$

where each token ${H}_{seq}^{j}$ has the same dimensionality as the LLM’s word embeddings.

The model predicts the response ${X}_{response}$ autoregressively, with the modality encoder and generative LLM remain frozen, and only the parameters from projection module $\theta =\{{W}_{proj}\}$ are updated. The optimization objective is given by:

$$P\left({X}_{response}|{X}_{instruction},{X}_{seq}\right)=\prod\limits_{i=1}^{L}{p}_{\theta }\left({x}_{i}|{X}_{instruction},{X}_{seq}\right)$$

### Instruction tuning

To improve OPUS-PLLM’s multi-task performance and instruction-following capabilities, we employ simultaneous fine-tuning of both: 1) the generative LLM using Low-Rank Adaptation (LoRA) [53], and 2) the projection module. This optimization is also performed using the OPUS-InstructionCorpus dataset.

In this process, the pre-trained weight matrix ${W}_{llm}$ of the generative LLM is frozen, while the low-rank matrices $A$ and $B$ are designated as trainable parameters. Matrix $A$ determines the rank and consequently influences the magnitude of weight adjustments, whereas matrix $B$ is configured with dimensions aligned to the output dimensions of the target layer. This approach facilitates efficient model training by focusing optimization efforts solely on $A$ and $B$, thereby circumventing the necessity for extensive fine-tuning of a vast array of parameters.

Additionally, the parameters from the projection module ${W}_{proj}$ are trainable but with a relatively small learning rate. This allows the projection module to adapt to the language model’s paradigm while preserving its ability to map protein sequence information into the language space. While maintaining the same objective function as the modality refinement step, this stage operates with a distinct set of trainable parameters $\theta =\{A,B,{W}_{proj}\}$.

### Datasets

#### Training set for each step in OPUS-PLLM

For the modality encoding step, we use the ProtDescribe [9], a dataset comprising 546,026 protein sequence-functional description pairs, as our training set. The protein descriptions in it are sourced from SWISS-PROT [54], a high-quality, annotated subset of the UniProt database [17]. The dataset is split into three subsets: 436,822 pairs for the training set, 54,602 pairs for the validation set, and 54,602 pairs for the test set.

For the modality refinement and instruction tuning steps, we develop OPUS-InstructionCorpus through a structured four-stage pipeline using SWISS-PROT 2022–01 as our foundational training data: 1) data extraction, 2) data relabeling, 3) data augmentation, and 4) data merging and homology reduction.

The data extraction step involves retrieving relevant entries from SWISS-PROT 2022–01 that meet our training criteria. Specifically, we extract high-confidence non-empty annotations across five key categories: GO term, UniProt keyword, functional description, protein family, and subcellular localization. For EC number, we directly adopt the pre-processed training and testing sequence splits from CLEAN [28].

The data relabeling step addresses label inconsistency in subcellular localization annotations from the data extraction stage. To ensure fair evaluation of language models on this task, we adopt DeepLoc’s annotation rules [27], which classify proteins into 10 standardized classes**:** “Membrane”, “Cytoplasm”, “Reticulum”, “Apparatus”, “Lysosome/Vacuole”, “Mitochondrion”, “Nucleus”, “Peroxisome”, “Plastid” and “Extracellular”. However, SWISS-PROT labels often deviate from this schema, either by providing overly specific terms (e.g., “basolateral” instead of “Membrane”) or alternative naming conventions (e.g., “chloroplast” instead of “Plastid”). To unify the labeling system, we implement a two-step LLM-assisted pipeline: 1) Utilize the LLM assesses whether a SWISS-PROT label can be mapped to one of the 10 DeepLoc classes. 2) For remaining entries, the LLM assigns the closest matching DeepLoc label. Finally, we manually validate the LLM-processed labels using regular expressions, removing any non-compliant samples to ensure dataset consistency.

The data augmentation step aims at enhancing instruction diversity to improve LLM performance. To ensure a diverse and sufficiently large instruction pool, we employ LLM-based paraphrasing to expand predefined task instructions while preserving semantic consistency. The process is as follows: 1) Seed instruction creation. For each task, we manually curate a preliminary instruction pool containing 7–20 distinct templates, each phrased to elicit task-relevant responses. 2) LLM-based paraphrasing. Using GLM4-plus [40], we systematically rephrase the seed instructions to generate diverse variants while retaining original intent. 3) Instruction assignment. Each sequence-response pair is paired with a suitable instruction by random sampling from its task-specific pool. The final dataset comprises 509 K unique textual instructions. Meanwhile, we replace raw EC numbers (e.g., “1.1.1.1”) with standardized enzyme names (e.g., “alcohol dehydrogenase”) as LLMs process natural language enzyme names more effectively than numeric codes.

The data merging and homology reduction step merges all six sub-datasets from previous steps while implementing homology controls. Using blastp [21], we remove sequences exhibiting > 85% identity to any benchmark entry across all 17 testing datasets with the e-value set at 1e-3. This filtering yields OPUS-InstructionCorpus, a comprehensive instruction dataset containing 2.11M unique instruction-sequence-response triples spanning six protein functional annotations.

#### Protein function prediction benchmarks

The benchmarks employed in this study incorporate 17 testing datasets covering five protein function prediction tasks: subcellular localization, GO term prediction, UniProt keyword prediction, EC number prediction and functional description generation.

The four Swiss2024-series testing datasets constructed in this study are derived from SWISS-PROT 2024–05 (released November 2024), containing the most up-to-date curated protein annotations. Following the pipeline established for OPUS-InstructionCorpus (including data extraction, relabeling, and merging), we respectively develop Swiss2024_Loc which includes 904 sequences for subcellular localization, Swiss2024_GO which includes 1,607 sequences for GO term prediction, Swiss2024_Keyword which includes 1,712 sequences for UniProt keyword prediction, and Swiss2024_Description: 1,687 sequences for functional description generation.

In addition, our evaluation incorporates 13 testing datasets formatted in other studies, including OPILoc [35], DeepLoc [27], EC_New392 [28], EC_Price149 [28], three CASPSimilar-series datasets, three IDFilter-series datasets, and three Uniprot-series datasets. CASPSimilar, IDFilter, and Uniprot-series datasets are generated by applying sequence-level deduplication to their original OPI versions [35]. Below are the construction details regarding the CASPSimilar, IDFilter, and Uniprot-series datasets:

The CASPSimilar-series datasets are constructed from 184 candidate sequences originally identified in OPI’s benchmark through BLAST searches against the SWISS-PROT-all database, selecting sequences with > 50% identity to any of 51 CASP14 target sequences. After applying sequence-level deduplication to eliminate redundancy, the three final curated datasets each comprise 130 protein sequences. These sequences are annotated with GO term, UniProt keyword, and functional description, respectively.

The IDFilter-series datasets originally include 1,112 sequences sourced from OPI’s benchmark. The OPI team first excludes sequences belonging to the CASPSimilar-series datasets, then clusters the remaining sequences using CD-HIT with a sequence identity threshold of ≥ 80%. From these clusters, they randomly sample all sequences from 500 distinct groups. In this study, we also deduplicate the dataset at the sequence level, resulting in three final IDFilter-series datasets, each comprising 922 protein sequences.

The UniProt-series datasets are constructed by randomly sampling 1% of the protein sequences from OPI’s training dataset, with subsequent exclusion of any sequences overlapping with the CASPSimilar-series and IDFilter-series datasets. This initial sampling yields 4,562 sequences, which are then deduplicated at the sequence level in this study. The final three UniProt-series datasets each contain 3515 protein sequences.

#### Training details

For the modality encoding step, we employ the Adam optimizer with a fixed learning rate of 1e-4 and L2 weight decay (λ = 0.001). The temperature parameter (τ) is set at 0.007. Training is conducted for 100 epochs using a batch size of 128 on a single NVIDIA A100-80G GPU.

For the modality refinement step, we freeze all parameters in both generative LLM and modality encoder, training only the multi-head projection module. We employ the AdamW optimizer with a fixed learning rate of 4e-4. Training is executed over a single epoch using four NVIDIA A100-80G GPUs, with each GPU processing a batch size of 8 (totaling 32 samples across all GPUs) and a maximum input sequence length of 256 tokens. To maximize training efficiency and minimize memory consumption, the framework employs FP16 mixed-precision training. Additionally, DeepSpeed ZeRO Stage 3 is integrated to dynamically partition model parameters, gradients, and optimizer states across GPUs.

For the instruction tuning step, we utilize Low-Rank Adaptation (LoRA) for efficient fine-tuning the base LLM. The LoRA configuration employs a rank of 64, scaling factor (α) of 16, and dropout rate of 0.05, while the AdamW optimizer is used with distinct learning rates (4e-4 for the LLM, 1e-5 for projection module). A cosine learning rate scheduler with a 0.01 warmup ratio is applied, and the model is trained for 2 epochs on four NVIDIA A100-80G GPUs using a per-GPU batch size of 6 (totaling 24 samples across all GPUs). To maximize hardware efficiency and reduce memory overhead, FP16 mixed-precision training and DeepSpeed ZeRO Stage 3 [55] are also adopted.

For training task-specific discriminative models, we process PLM-derived representations through an optimized MLP-head architecture adapted from OPUS-GO [32]. All models are trained using the Adam optimizer (fixed learning rate = 1e-3) for 30,000 steps with a batch size of 64 on a single NVIDIA A100-80G GPU. To prevent overfitting and ensure generalization, we implement early stopping with a patience interval of 4 evaluations, selecting the optimal model checkpoint based on maximum validation performance, assessed every 2,000 training steps.

#### Performance metrics

In this study, we employ task-specific metrics: accuracy for single-label subcellular localization prediction and multiple-choice question answering (MCQA) task averaged at the protein level; precision-recall-F1 scores for multi-label tasks (GO term, UniProt keyword, EC number) that also averaged at the protein level. For evaluating functional description generation, we employ nine established metrics: 1) ROUGE variants, including ROUGE-1 (R1, unigram recall), ROUGE-2 (R2, bigram overlap), ROUGE-L (RL, sentence-level longest common subsequence), and ROUGE-Lsum (RLS, document-level longest common subsequence across concatenated references), quantify lexical alignment; 2) BLEU (B) measures n-gram precision with a brevity penalty for length normalization; 3) BERTScore variants, including BP (precision), BR (recall), and BF (F1-score), are computed using BioBERT-large-cased-v1, a biomedical domain-specific pretrained model developed by Lee et al. [56], capturing semantic relevance in the context of protein; 4) METEOR (M) provides a balanced evaluation via exact/stem/synonym matches with word order penalties. All metrics are computed at the corpus level, using standardized tokenization from Huggingface’s evaluate library. A detailed description of the evaluation metrics used in this study is provided in Additional file 2.

### Detailed descriptions of subtasks

#### UniProt keyword prediction

This task focuses on identifying standardized annotations associated with the function, structure, or biological processes of a given protein sequence. Keywords in the UniProt database encapsulate the core characteristics of proteins, such as “Enzyme activity”, “Membrane protein”, or “DNA-binding protein”, which directly reflect their functional, structural, or biological attributes. Predicting these keywords enables researchers to rapidly grasp the functional profile of an uncharacterized protein, offering actionable guidance for functional annotation, proteomic analysis, and downstream biological research.

#### Functional description generation

This task aims to generate natural language descriptions of a protein’s biological functions, structural features, and cellular/organismal roles using only its amino acid sequence. Unlike discrete labels or keywords, functional descriptions deliver richer, more detailed, and human-readable information, including the protein’s molecular mechanisms, involved biological processes, and contextual research background [14, 34]. This approach not only accelerates researchers’ comprehension of protein function but also underpins knowledge integration, biological information mining, and downstream bioinformatics analyses.

#### Subcellular localization prediction

Aberrant subcellular localization of proteins disrupts their normal function and is implicated in numerous human diseases, including cardiovascular disorders and cancer. Thus, determining a protein’s subcellular localization yields critical insights into cellular signaling mechanisms, biomolecular interactions, and holds substantial implications for drug target discovery.

#### GO term prediction

Gene Ontology (GO) terms is a universally accepted framework for functional classification of biological sequences, which plays a pivotal role in protein biology research, including functional annotation of protein domains and integration of proteomic data across diverse organisms. Notably, GO terms are interoperable with numerous other biomedical ontologies, establishing a foundational framework for the application of computational methods in biomedical research.

#### EC number prediction

The Enzyme Commission (EC) number is a standardized classification system for categorizing the catalytic reaction specificities of enzymes. Accurate EC number prediction for enzymes not only elucidates their catalytic functions and roles within metabolic networks but also advances research in metabolic engineering, drug target identification, and systems biology.

All the datasets used in this study are listed in Additional file 1: Table S16.

## Supplementary Information

Additional file 1: Table S1. Detailed comparison of OPUS-PLLM versus state-of-the-art generative LLMs on: three UniProt keyword datasets, three GO term datasets, and two EC number datasets. Table S2. Some examples illustrating subcellular localization prediction generated by OPUS-PLLM and baseline biological-knowledge-integrated generative LLMs. Table S3. Some examples illustrating GO term prediction generated by OPUS-PLLM and baseline biological-knowledge-integrated generative LLMs. Table S4. Some examples illustrating UniProt keyword prediction generated by OPUS-PLLM and baseline biological-knowledge-integrated generative LLMs. Table S5. Some examples illustrating EC number prediction generated by OPUS-PLLM and baseline biological-knowledge-integrated generative LLMs. Table S6. Detailed comparison of OPUS-PLLM versus discriminative approaches based on different PLM representationson: three UniProt keyword datasets, three GO term datasets, and two EC number datasets. Table S7. Detailed comparison of OPUS-PLLM versus state-of-the-art generative LLMs on two Swiss2024-series testing datasets. Table S8. Detailed comparison of OPUS-PLLM versus discriminative approaches based on different PLM representationson two Swiss2024-series testing datasets. Table S9. Detailed performance comparison of OPUS-PLLM and its four ablated variantson: four GO term datasets, four UniProt keyword datasets and two EC number datasets. Table S10. Detailed performance comparison of discriminative models using different representations. Table S11. Performance comparison between OPUS-PLLM and leading general-purpose large language modelswith and without retrieval-augmented generationacross subcellular localization prediction, GO term prediction, UniProt keyword prediction, and EC number prediction. Table S12. Performance comparison between OPUS-PLLM and leading general-purpose large language modelswith and without retrieval-augmented generationon functional description generation. Table S13. Comparison of OPUS-PLLM against several leading discriminative models. Table S14. Performance comparison between OPUS-PLLM and DeepGO-SE on the DeepGO-SE test set. OPUS-PLLM is trained using the same data as DeepGO-SE, with the BP, MF, and CC datasets combined into a unified dataset. Table S15. Comparison of mean Matthews Correlation Coefficientand low-frequency-term MCCfor generativeand discriminative models utilizing ESM2 representations. Table S16. Summary of datasets used for benchmarking OPUS-PLLM. Fig S1. Performance of OPUS-PLLM across different GO term depths.

Additional file 2. Detailed descriptions of evaluation metrics.

## Data Availability

The OPUS-PLLM source code is available on GitHub [57] (https://github.com/Fanchuana/OPUS-PLLM) under the GNU General Public License v3.0(GPL-3.0). The datasets (OPUS-InstructionCorpus [58], OPUS-InstructionCorpus-Evol [59], and Benchmark [60]) are deposited in Hugging Face (https://huggingface.co/collections/YifanXu24/opus-pllm-dataset) under the Creative Commons Attribution 4.0 International (CC BY 4.0) license. Meanwhile, we also provided the pretrained models [61] at Hugging Face (https://huggingface.co/collections/YifanXu24/opus-pllm).
