# Supplementary material for: Advancing generative large language models toward discriminative performance in protein function prediction
Source: Genome Biol. 2026 May 21;27:226. doi: 10.1186/s13059-026-04109-8 (PMC13366677; doi:10.1186/s13059-026-04109-8)
Supplement: Supplementary file 1 — Additional file 1: Table S1. Detailed comparison of OPUS-PLLM versus state-of-the-art generative LLMs on: three UniProt keyword datasets, three GO term datasets, and two EC number datasets. Table S2. Some examples illustrating subcellular localization prediction generated by OPUS-PLLM and baseline biological-knowledge-integrated generative LLMs. Table S3. Some examples illustrating GO term prediction generated by OPUS-PLLM and baseline biological-knowledge-integrated generative LLMs. Table S4. Some examples illustrating UniProt keyword prediction generated by OPUS-PLLM and baseline biological-knowledge-integrated generative LLMs. Table S5. Some examples illustrating EC number prediction generated by OPUS-PLLM and baseline biological-knowledge-integrated generative LLMs. Table S6. Detailed comparison of OPUS-PLLM versus discriminative approaches based on different PLM representationson: three UniProt keyword datasets, three GO term datasets, and two EC number datasets. Table S7. Detailed comparison of OPUS-PLLM versus state-of-the-art generative LLMs on two Swiss2024-series testing datasets. Table S8. Detailed comparison of OPUS-PLLM versus discriminative approaches based on different PLM representationson two Swiss2024-series testing datasets. Table S9. Detailed performance comparison of OPUS-PLLM and its four ablated variantson: four GO term datasets, four UniProt keyword datasets and two EC number datasets. Table S10. Detailed performance comparison of discriminative models using different representations. Table S11. Performance comparison between OPUS-PLLM and leading general-purpose large language modelswith and without retrieval-augmented generationacross subcellular localization prediction, GO term prediction, UniProt keyword prediction, and EC number prediction. Table S12. Performance comparison between OPUS-PLLM and leading general-purpose large language modelswith and without retrieval-augmented generationon functional description generation. Table S13. Com [file 13059_2026_4109_MOESM1_ESM.pdf]

Additional file 1:

Advancing Generative Large Language Models Toward Discriminative Performance in Protein Function Prediction

Lv *et al*.

Table S1. Detailed comparison of OPUS-PLLM versus state-of-the-art generative LLMs on: three UniProt keyword datasets, three GO term datasets, and two EC number datasets.

Benchmarks	Models	Precision	Recall	F1-Score	MCC
	Gene Ontology (GO) Term				
CASPSimilar_GO	OPI-Galactica-6.7B	0.697	0.678	0.677	0.198
	OPI-Llama3-8B	0.109	0.087	0.092	0.091
	OPUS-PLLM-Llama3-8B	**0.846**	**0.863**	**0.848**	**0.347**
IDFilter_GO	OPI-Galactica-6.7B	0.704	0.694	0.685	0.217
	OPI-Llama3-8B	0.676	0.671	0.653	0.293
	OPUS-PLLM-Llama3-8B	**0.830**	**0.805**	**0.801**	**0.444**
UniProt_GO	OPI-Galactica-6.7B	0.717	0.691	0.687	0.177
	OPI-Llama3-8B	0.697	0.685	0.673	0.247
	OPUS-PLLM-Llama3-8B	**0.835**	**0.806**	**0.801**	**0.391**
	UniProt Keyword				
CASPSimilar_Keyword	OPI-Galactica-6.7B	0.754	0.693	0.714	0.335
	OPI-Llama3-8B	0.417	0.481	0.422	0.261
	OPUS-PLLM-Llama3-8B	**0.884**	**0.859**	**0.862**	**0.666**
IDFilter_Keyword	OPI-Galactica-6.7B	0.816	0.788	0.789	0.478
	OPI-Llama3-8B	0.754	0.779	0.747	0.543
	OPUS-PLLM-Llama3-8B	**0.905**	**0.860**	**0.871**	**0.780**
UniProt_Keyword	OPI-Galactica-6.7B	0.833	0.795	0.800	0.455
	OPI-Llama3-8B	0.793	0.804	0.783	0.558
	OPUS-PLLM-Llama3-8B	**0.922**	**0.877**	**0.889**	**0.748**
	Enzyme Commission (EC) Number				
EC_Price149	OPI-Galactica-6.7B	0.027	0.027	0.027	0.016
	OPI-Llama3-8B	0.074	0.074	0.074	0.052
	OPUS-PLLM-Llama3-8B	**0.262**	**0.262**	**0.262**	**0.314**
EC_New392	OPI-Galactica-6.7B	0.270	0.266	0.260	0.128
	OPI-Llama3-8B	0.372	0.337	0.347	0.155
	OPUS-PLLM-Llama3-8B	**0.495**	**0.479**	**0.482**	**0.452**

Table S2. Some examples illustrating subcellular localization prediction generated by OPUS-PLLM and baseline biological-knowledge-integrated generative LLMs.

Subcellular Localization - Example 1	
Sequence	AGFPEQEPEPKFWNDWAQKTLDKALSLQTLNKNKAQNLILFLGDGMGVPTV<br>TAARILKGQLRGQPGEEGQLEMDKFPFVALSKTYNTNAQVADSAGTATAYLC<br>GVKANEGTVGVSAAAVRSQANTTQGNEVTSILRWAKDAGKSIGIVTTTRVNH<br>ATPSAAYAHCVDRDWYSDNEMPADAVEAGCKDIARQLFENIPDIDVIMGGGR<br>KYMYPKNTTDVEYPGQPKHSGTRKDGRNLVKEWVDRNTEKKGHYVWNKK<br>DLLSLNPTKVDYLLGLFEPADLPYDLERNKETDPSLSEMVEVAIKILRRNPNG<br>FYLLVEGGRIDHGHHEGKDKQAIHEAVEMDRAIGRADLMTSTSDTLTVVTAD<br>HSHLFSFGGYTPRGNEIFGLAAFISDVDQKPFTAILYGNGPGYKLVNGARENV<br>STVDYQDNSYLAQAAVPLSSETHGGEDVAVFAKGPMAHLLHGVHEQNYIPH<br>AMAYAACIGQNR
Instruction	Through sequence analysis, forecast the protein's subcellular positioning in eukaryotic cells.
Ground truth	Extracellular
Prot2Text	/--/
BioMedGPT	Cytoplasm.
InstrucProtein	The protein is located in extracellular exosome.
OPI-Galactica-6.7B	Extracellular
OPI-Llama3-8B	Extracellular
OPUS-PLLM-Llama3-8B	Extracellular
**Subcellular Localization - Example 2**	
Sequence	MSEEPPSDQVNSLRDSLNRWNQTRQQNSQGFNESAKTLFSSWADSLNTRAQ<br>DIYQTLPVSRQDLVQDQEPSWFQLSRTERMVLFVCFLLGATACFTLCTFLFPV<br>LAAKPRKFGLLWTMGSLLFVLAFGVLMGPLAYLKHLTARERLPFSMFFFAT<br>CFMTIYFAAFSKNTVLTITCALLELVAVIYYAISYFPFGATGLRMLSSAGVNS<br>ARGVLRI
Instruction	Utilize the protein sequence to make projections about the subcellular distribution within eukaryotic cells.
Ground truth	apparatus
Prot2Text	/--/
BioMedGPT	Cytoplasm.
InstrucProtein	The protein is located in golgi membrane.
OPI-Galactica-6.7B	reticulum
OPI-Llama3-8B	reticulum
OPUS-PLLM-Llama3-8B	apparatus.

Table S3. Some examples illustrating GO term prediction generated by OPUS-PLLM and baseline biological-knowledge-integrated generative LLMs.

Gene Ontology (GO) Term - Example 1	
Sequence	MPYFAQRLYNTCKASFSSDGPITEDALEKVRNVLEKIKPSDVGIEQDAQLA RSRSGPLNERNGSNQSPPAIKYLHLHECDSFSIGIFCMPPSSMIPLHNHPGMT VLSKLVYGSMHVKSYDWLEPQLTEPEDPSQARPAKLVKDTEMTAQSPVTT LYPKSGGNIHCFKAITHCAILDILAPPYSSEHDRHCTYFRKSRREDLPGELEV DGEVVTDVTWLEEFQPPDDFVIRRIPYRGPVIRT
Instruction	Given a protein sequence, predict the corresponding Gene Ontology term that describes its molecular function, biological process, and cellular component.
Ground truth	cytoplasm ; nucleus ; cysteine dioxygenase activity ; iron ion binding ; cellular response to hypoxia ; detection of hypoxia ; peptidyl-cysteine oxidation
Prot2Text	/--/
BioMedGPT	Belongs to the cytochrome P450 family.
InstrucProtein	Based on the record, the answer is no.
OPI-Galactica-6.7B	cytoplasm; nucleus; 2 iron, 2 sulfur cluster binding; metal ion binding; oxidoreductase activity; quinone binding
OPI-Llama3-8B	cytoplasm; nucleus; MAP kinase tyrosine/serine/threonine phosphatase activity; myosin phosphatase activity; protein tyrosine phosphatase activity; protein dephosphorylation
OPUS-PLLM-Llama3-8B	cytosol; nucleus; cysteine dioxygenase activity; iron ion binding; cellular response to hypoxia; detection of hypoxia; response to hypoxia
Gene Ontology (GO) Term - Example 2	
Sequence	MPYFAQRLYNTCKASFSSDGPITEDALEKVRNVLEKIKPSDVGIEQDAQLA RSRSGPLNERNGSNQSPPAIKYLHLHECDSFSIGIFCMPPSSMIPLHNHPGMT VLSKLVYGSMHVKSYDWLEPQLTEPEDPSQARPAKLVKDTEMTAQSPVTT LYPKSGGNIHCFKAITHCAILDILAPPYSSEHDRHCTYFRKSRREDLPGELEV DGEVVTDVTWLEEFQPPDDFVIRRIPYRGPVIRT
Instruction	List all gene ontology labels for this protein.
Ground truth	membrane ; ATP binding ; ATP hydrolysis activity ; copper ion binding ; P-type monovalent copper transporter activity ; detoxification of copper ion ; response to copper ion
Prot2Text	/--/
BioMedGPT	Probable copper-transporting ATPase.
InstrucProtein	Based on the record, the answer is no.
OPI-Galactica-6.7B	chloroplast membrane; membrane; plasma membrane; ferrous iron transmembrane transporter activity; iron ion transmembrane transporter activity; iron ion transmembrane transport
OPI-Llama3-8B	chloroplast; chloroplast membrane; chloroplast thylakoid membrane; cytosol; membrane; plasma membrane; plastid; thylakoid; thylakoid membrane; ATP binding; ATP hydrolysis activity; copper ion binding; P-type monovalent copper

	transporter activity; copper ion homeostasis; copper ion import; copper ion transport; intracellular copper ion homeostasis; response to cadmium ion; response to copper ion; response to silver ion
OPUS-PLLM-Llama3-8B	membrane; plasma membrane; ATP binding; ATP hydrolysis activity; copper ion binding; P-type divalent copper transporter activity; P-type monovalent copper transporter activity; copper ion homeostasis

Table S4. Some examples illustrating UniProt keyword prediction generated by OPUS-PLLM and baseline biological-knowledge-integrated generative LLMs.

UniProt Keyword - Example 1	
Sequence	MSKKILFLIKVIVFIFTTFSLPLYAEKIRDLTSIQGIRDNPLIGYGLIVGLDGTGDQ STQAPFTNQSLKNMLSQLGVSIPSNTNMNTKNVAAVIVTANLPPFSHAGEKID VVVSSMGNARSLKGGTLLMTPLKGANNQIYAIAQGNILVSEKNNKKSKIHNF YSNQVNSGKIHHGATIEREIDNNFGKQKTINLQLNQENFSTAQRISDMINTKYP DTATPINSKTVQLNTSANNDVQVHMLSNIQDIDISLPSQEAKVVVNSRTGSIVI NQSVRLGSCVVSNGNMSIIVHQIKNKKRDLYFLKSFNKNIKKDQSIEDMADKN YMNDIATNKENLHNIVRALNKLGTKPDELMSILQLMKSAGCLNAKLEIV
Instruction	Analyze the provided protein sequence and predict the UniProtKB keywords associated with it.
Ground truth	Bacterial flagellum ; Reference proteome ; Signal
Prot2Text	/--/
BioMedGPT	Belongs to the basal body.
InstrucProtein	Based on the record, the answer is no.
OPI-Galactica-6.7B	Bacterial flagellum; Periplasm; Signal
OPI-Llama3-8B	Cell outer membrane; Lipoprotein; Membrane; Palmitate; Signal; Transmembrane; Transmembrane beta strand
OPUS-PLLM-Llama3-8B	Bacterial flagellum; Periplasm; Reference proteome; Signal
UniProt Keyword - Example 2	
Sequence	MATKLLSLTCIRKERFSERYPLVRKHLTRSRDGGGGSSSETAAFEIDDPISRAVF QVLGMTCSACAGSVEKAIKRLPGIHDAVIDALNNRAQILFYPNSVDVETIRETI EDAGFEASLIENEANERSRQVCRIRINGMTCTSCSSTIERVLQSVNGVQRAHVA LAIEEAEIHYDPRLSSYDRLLEEIENAGFEAVLISTGEDVSKIDLKIDGELTDES MKVIERSLEALPGVQSVEISHGTDKISVLYKPDVTGPRNFIQVIESTVFGHSGHI KATIFSEGGVGRESQKQGEIKQYYKSFLWSLVFTVPVFLTAMVFMYIPGIKDL LMFKVINMLTVGEIIRCVLATPVQFVIGWRFYTGSYKALRRGSANMDVLIALG TNAAYFYSLYTVLRAATSPDFKGVDFFETSAMLISFIILGKYLEVMAKGKTSQ AIAKLMNLAPDTAILLSLDKEGNVTGEEEIDGRLIQKNDVIKIVPGAKVASDGY VIWGQSHVNESMITGEARPVAKRKGDTVIGGTLNENGVLHVKVTRVGSESAL AQIVRLVESAQLAKAPVQKLADRISKFFVPLVIFLSFSTWLAWFLAGKLHWYP ESWIPSSMDSFELALQFGISVMVIACPCALGLATPTAVMVGTGVGASQGVLIK GGQALERAHKVNCIVFDKTGTLTMGKPVVVKTKLLKNMVLREFYELVAATE VNSEHPLAKAIVEYAKKFRDDEENPAWPEACDFVSITGKGVKATVKGREIMV GNKNLMNDHKVIIPDDAEELLADSEDMAQTGILVSINSELIGVLSVSDPLKPSA REAISILKSMNIKSIMVTGDNWGTANSIAREVGIDSVIAEAKPEQKAEKVKELQ AAGHVVAMVGDGINDSPALVAADVGMAIGAGTDIAIEAADIVLMKSNLEDVI TAIDLSRKTFSRIRLNYVWALGYNLMGIPIAAGVLFPGTRFRLPPWIAGAAMA ASSVSVVCCSLLLKNYKRPKKLDHLEIREIQVERV

Instruction	Give all UniprotKB based on this protein sequence.
Ground truth	ATP-binding ; Copper ; Copper transport ; Ion transport ; Magnesium ; Membrane ; Metal-binding ; Nucleotide-binding ; Reference proteome ; Repeat ; Translocase ; Transmembrane ; Transmembrane helix ; Transport
Prot2Text	/--/
BioMedGPT	Belongs to the cation transport ATPase (P-type) (TC 3.A.3) family. Type IB subfamily.
InstrucProtein	Based on the record, the answer is no.
OPI-Galactica-6.7B	membrane; plasma membrane; ATP binding; ATP hydrolysis activity; metal ion binding; P-type divalent copper transporter activity; copper ion import across plasma membrane
OPI-Llama3-8B	ATP-binding cassette (ABC) transporter complex; membrane; plasma membrane; ATP binding; ATP hydrolysis activity; ATPase-coupled transmembrane transporter activity; metal ion binding; transmembrane transporter activity; transmembrane transport
OPUS-PLLM-Llama3-8B	ATP-binding; Copper; Copper transport; Ion transport; Magnesium; Membrane; Metal-binding; Nucleotide-binding; Phosphoprotein; Reference proteome; Repeat; Translocase; Transmembrane; Transmembrane helix; Transport RNA-binding; Viral immunoevasion

Table S5. Some examples illustrating EC number prediction generated by OPUS-PLLM and baseline biological-knowledge-integrated generative LLMs.

Enzyme Commission (EC) Number - Example 1	
Sequence	MTAQVSSEASHAETLQDEVLAEVRNHIGHLTLNRPAGLNAITLNMVRRLASQ LKAWADDPQVYAVVLRGAGEKAFCAGGDIRSLYDSFKNGDTLHQDFFVEEY ALDLAIHHYRKPVLALMDGFVLGGGMGLVQGADLRVVTERSRLAMPEVAIG YFPDVGGSYFLPRIPGELGIYLGVTGVQIRAADALYCGLADWYLESSKLADLD NKLDRLQWHDSPLKDLQGVLAKLAVQQLPDAPLAVLRPAIDHFFALPDVPSIV EQLQQVTVADSHEWALTTAHLMQTRSPLAMAVTLEMLRRGRRLPLEQCFAL ELHLDRQWFERGDLIEGVRALIIDKDKAPRWNPPTLHGLALSHVESFFHNFKK VAN
Instruction	Return the EC number of the protein sequence.
Ground truth	3-hydroxyisobutyryl-CoA hydrolase (3.1.2.4)
Prot2Text	/--/
BioMedGPT	Belongs to the enoyl-CoA hydratase/isomerase family.
InstrucProtein	Based on the record, the answer is no.
OPI-Galactica-6.7B	1.14.11.41(L-arginine hydroxylase)
OPI-Llama3-8B	6.3.2.2(glutamate---cysteine ligase)
OPUS-PLLM-Llama3-8B	3-hydroxyisobutyryl-CoA hydrolase
Enzyme Commission (EC) Number - Example 2	
Sequence	MPDKKPGLRSAQWFGTADKNGFMYRSWMKNQGIADHQFHGKPIIGICNTWS ELTPCNAHFRQIAEHVKRGVIEAGGFPVEFPVFSNGESNLRPTAMLTRNLASM DVEEAIRGNPIDGVVLLTGCDKTTPALLMGAASCDVPAIVVTGGPMLNGKHK GQDIGSGTVVWQLSEQVKAGTITLDDFLAAEGGMSRSAGTCNTMGTASTMA CMAEALGTSLPHNAAIPAVDARRYVLAHMSGMRAVEMVREDLKLSKILTKE AFENAIRVNAAIGGSTNAVIHLKAIAGRIGVELDLDDWTRIGRGMPTIVDLQPS GRFLMEEFYYAGGLPAVLRRLGEANLIPHPNALTVNGKSLGENTQDSPIYGQD EVIRTLDNPIRADGGICVLRGNLAPLGAVLKPSAASPALMQHRGRAVVFENFD MYKARINDPELDVDANSILVMKNCGPKGYPGMAEVGNMGLPAKLLAQGVT DMVRISDARMSGTAYGTVVLHVAPEAAAGGPLATVKEGDWIELDCANGRLH LDIPDAELAARMADLAPPQKLIVGGYRQLYIDHVLQADQGCDFDFLVGCRGA EVPRHSH
Instruction	Please provide the Enzyme Commission of this protein.
Ground truth	L-arabinonate dehydratase (5.1.3.3)
Prot2Text	/--/
BioMedGPT	Belongs to the IlvD/Edd family.
InstrucProtein	Based on the record, the answer is no.
OPI-Galactica-6.7B	4.2.1.1(carbonic anhydrase)
OPI-Llama3-8B	1.2.7.3(2-oxoglutarate synthase)

OPUS-PLLM-Llama3-8B	L-arabinonate dehydratase

Table S6. Detailed comparison of OPUS-PLLM versus discriminative approaches based on different PLM representations (ESM2, ProtT5, Ankh) on: three UniProt keyword datasets, three GO term datasets, and two EC number datasets.

Benchmarks	Models	Precision	Recall	F1-Score	MCC
Gene Ontology (GO) Term					
CASPSimilar_GO	ESM2	0.847	0.778	0.796	0.165
	ProtT5	**0.850**	0.767	0.785	0.258
	Ankh	0.810	0.653	0.680	0.254
	OPUS-PLLM-Llama3-8B	0.846	**0.863**	**0.848**	**0.347**
IDFilter_GO	ESM2	0.823	0.699	0.725	0.329
	ProtT5	0.826	0.725	0.737	0.372
	Ankh	0.807	0.679	0.707	0.380
	OPUS-PLLM-Llama3-8B	**0.830**	**0.805**	**0.801**	**0.444**
UniProt_GO	ESM2	**0.845**	0.698	0.730	0.290
	ProtT5	0.834	0.720	0.741	0.341
	Ankh	0.811	0.660	0.693	0.335
	OPUS-PLLM-Llama3-8B	0.835	**0.806**	**0.801**	**0.391**
UniProt Keyword					
CASPSimilar_Keyword	ESM2	0.873	0.824	0.834	0.324
	ProtT5	**0.902**	0.804	0.837	0.500
	Ankh	0.881	0.706	0.765	0.487
	OPUS-PLLM-Llama3-8B	0.884	**0.859**	**0.862**	**0.666**
IDFilter_Keyword	ESM2	**0.923**	0.829	0.857	0.740
	ProtT5	0.918	0.849	0.867	0.694
	Ankh	0.920	0.854	0.871	0.736
	OPUS-PLLM-Llama3-8B	0.905	**0.860**	**0.871**	**0.780**
UniProt_Keyword	ESM2	0.928	0.833	0.863	0.707
	ProtT5	**0.935**	0.856	0.880	0.679
	Ankh	0.930	0.854	0.877	0.723
	OPUS-PLLM-Llama3-8B	0.922	**0.877**	**0.889**	**0.748**
Enzyme Commission (EC) Number					
EC_Price149	ESM2	**0.267**	**0.299**	**0.275**	0.217
	ProtT5	0.242	0.275	0.252	0.221
	Ankh	0.200	0.248	0.216	0.201
	OPUS-PLLM-Llama3-8B	0.262	0.262	0.262	**0.314**
EC_New392	ESM2	0.470	0.470	0.465	**0.462**
	ProtT5	0.486	0.466	0.470	0.436
	Ankh	0.483	0.477	0.475	0.460
	OPUS-PLLM-Llama3-8B	**0.495**	**0.479**	**0.482**	0.452

Table S7. Detailed comparison of OPUS-PLLM versus state-of-the-art generative LLMs on two Swiss2024-series testing datasets.

Benchmarks	Models	Precision	Recall	F1-Score	MCC
Gene Ontology (GO) Term					
Swiss2024_GO	OPI-Galactica-6.7B	0.297	0.303	0.275	0.075
	OPI-Llama3-8B	0.301	0.311	0.281	0.098
	OPUS-PLLM-Llama3-8B	**0.481**	**0.489**	**0.453**	**0.245**
UniProt Keyword					
Swiss2024_Keyword	OPI-Galactica-6.7B	0.559	0.556	0.530	0.219
	OPI-Llama3-8B	0.562	0.561	0.534	0.308
	OPUS-PLLM-Llama3-8B	**0.717**	**0.691**	**0.677**	**0.519**

Table S8. Detailed comparison of OPUS-PLLM versus discriminative approaches based on different PLM representations (ESM2, ProtT5, Ankh) on two Swiss2024-series testing datasets.

Benchmarks	Models	Precision	Recall	F1-Score	MCC
Gene Ontology (GO) Term					
Swiss2024_GO	ESM2	0.552	0.317	0.369	0.170
	ProtT5	**0.562**	0.364	0.403	0.186
	Ankh	0.502	0.286	0.333	0.170
	OPUS-PLLM-Llama3-8B	0.481	**0.489**	**0.453**	**0.245**
UniProt Keyword					
Swiss2024_Keyword	ESM2	0.780	0.617	0.658	**0.525**
	ProtT5	**0.784**	0.636	0.675	0.487
	Ankh	0.778	0.630	0.664	0.512
	OPUS-PLLM-Llama3-8B	0.717	**0.691**	**0.677**	0.519

Table S9. Detailed performance comparison of OPUS-PLLM and its four ablated variants (i.e. without modality encoding, without modality refinement, without both modality encoding and refinement, and without instruction tuning) on: four GO term datasets, four UniProt keyword datasets and two EC number datasets.

Benchmarks	Models	Precision	Recall	F1-Score
	Gene Ontology (GO) Term			
CASPSimilar_GO	w/o modality encoding	0.841	**0.866**	0.845
	w/o modality refinement	0.803	0.807	0.799
	w/o modality encoding and refinement	0.808	0.804	0.801
	w/o instruction tuning	0.568	0.577	0.565
	OPUS-PLLM-Llama3-8B	**0.846**	0.863	**0.848**
IDFilter_GO	w/o modality encoding	0.781	0.783	0.761
	w/o modality refinement	0.771	0.766	0.748
	w/o modality encoding and refinement	0.773	0.763	0.749
	w/o instruction tuning	0.574	0.562	0.548
	OPUS-PLLM-Llama3-8B	**0.830**	**0.805**	**0.801**
UniProt_GO	w/o modality encoding	0.796	0.771	0.765
	w/o modality refinement	0.784	0.758	0.752
	w/o modality encoding and refinement	0.781	0.754	0.749
	w/o instruction tuning	0.603	0.572	0.569
	OPUS-PLLM-Llama3-8B	**0.835**	**0.806**	**0.801**
Swiss2024_GO	w/o modality encoding	0.445	0.467	0.421
	w/o modality refinement	0.424	0.448	0.401
	w/o modality encoding and refinement	0.413	0.438	0.390
	w/o instruction tuning	0.352	0.336	0.317
	OPUS-PLLM-Llama3-8B	**0.481**	**0.489**	**0.453**
	UniProt Keyword			
CASPSimilar_Keyword	w/o modality encoding	0.873	0.825	0.838
	w/o modality refinement	0.834	0.844	0.827
	w/o modality encoding and refinement	0.865	0.807	0.826
	w/o instruction tuning	0.755	0.795	0.762
	OPUS-PLLM-Llama3-8B	**0.884**	**0.859**	**0.862**
IDFilter_Keyword	w/o modality encoding	0.889	0.852	0.858
	w/o modality refinement	0.885	0.849	0.854
	w/o modality encoding and refinement	0.882	0.849	0.852
	w/o instruction tuning	0.771	0.774	0.757
	OPUS-PLLM-Llama3-8B	**0.905**	**0.860**	**0.871**
UniProt_Keyword	w/o modality encoding	0.902	0.856	0.867
	w/o modality refinement	0.888	0.852	0.857
	w/o modality encoding and refinement	0.884	0.853	0.855
	w/o instruction tuning	0.789	0.780	0.769
	OPUS-PLLM-Llama3-8B	**0.922**	**0.877**	**0.889**
Swiss2024_Keyword	w/o modality encoding	0.699	0.680	0.663
	w/o modality refinement	0.684	0.664	0.645

	w/o modality encoding and refinement	0.678	0.669	0.645
	w/o instruction tuning	0.594	0.585	0.562
	OPUS-PLLM-Llama3-8B	**0.717**	**0.691**	**0.677**
	Enzyme Commission (EC) Number			
EC_Price149	w/o modality encoding	0.208	0.208	0.208
	w/o modality refinement	0.228	0.228	0.228
	w/o modality encoding and refinement	0.181	0.181	0.181
	w/o instruction tuning	0.154	0.154	0.154
	OPUS-PLLM-Llama3-8B	**0.262**	**0.262**	**0.262**
EC_New392	w/o modality encoding	0.450	0.418	0.426
	w/o modality refinement	0.393	0.382	0.385
	w/o modality encoding and refinement	0.404	0.380	0.387
	w/o instruction tuning	0.211	0.206	0.207
	OPUS-PLLM-Llama3-8B	**0.495**	**0.479**	**0.482**

Table S10. Detailed performance comparison of discriminative models using different representations.

Benchmarks	Models	Precision	Recall	F1-Score
Gene Ontology (GO) Term				
CASPSimilar_GO	ESM2	0.847	0.778	0.796
	ProtT5	0.850	0.767	0.785
	Ankh	0.810	0.653	0.680
	OPUS-PLLM-Llama3-8B	**0.860**	**0.805**	**0.816**
IDFilter_GO	ESM2	0.823	0.699	0.725
	ProtT5	0.826	0.725	0.737
	Ankh	0.807	0.679	0.707
	OPUS-PLLM-Llama3-8B	**0.869**	**0.784**	**0.799**
UniProt_GO	ESM2	0.845	0.698	0.730
	ProtT5	0.834	0.720	0.741
	Ankh	0.811	0.660	0.693
	OPUS-PLLM-Llama3-8B	**0.894**	**0.809**	**0.827**
Swiss2024_GO	ESM2	0.552	0.317	0.369
	ProtT5	0.562	0.364	0.403
	Ankh	0.502	0.286	0.333
	OPUS-PLLM-Llama3-8B	**0.599**	**0.443**	**0.470**
UniProt Keyword				
CASPSimilar_Keyword	ESM2	0.873	0.824	0.834
	ProtT5	**0.902**	0.804	0.837
	Ankh	0.881	0.706	0.765
	OPUS-PLLM-Llama3-8B	0.855	**0.838**	**0.837**
IDFilter_Keyword	ESM2	0.923	0.829	0.857
	ProtT5	0.918	0.849	0.867
	Ankh	0.920	0.854	0.871
	OPUS-PLLM-Llama3-8B	**0.926**	**0.869**	**0.883**
UniProt_Keyword	ESM2	0.928	0.833	0.863
	ProtT5	0.935	0.856	0.880
	Ankh	0.930	0.854	0.877
	OPUS-PLLM-Llama3-8B	**0.942**	**0.882**	**0.900**
Swiss2024_Keyword	ESM2	0.780	0.617	0.658
	ProtT5	**0.784**	0.636	0.675
	Ankh	0.778	0.630	0.664
	OPUS-PLLM-Llama3-8B	0.768	**0.658**	**0.680**
Enzyme Commission (EC) Number				
EC_Price149	ESM2	0.267	0.299	0.275
	ProtT5	0.242	0.275	0.252
	Ankh	0.200	0.248	0.216
	OPUS-PLLM-Llama3-8B	**0.269**	**0.302**	**0.280**

EC_New392	ESM2	0.470	0.470	0.465
	ProtT5	0.486	0.466	0.470
	Ankh	0.483	0.477	0.475
	OPUS-PLLM-Llama3-8B	**0.506**	**0.498**	**0.498**

Table S11. Performance comparison between OPUS-PLLM and leading general-purpose large language models (LLMs) with and without retrieval-augmented generation (RAG) across subcellular localization prediction, GO term prediction, UniProt keyword prediction, and EC number prediction.

Benchmarks	Models	Accuracy	Precision	Recall	F1-Score
		Subcellular Localization			
DeepLoc	DeepSeek-V3	0.104	-	-	-
	DeepSeek-R1	0.254			
	Qwen3-235B	0.243	-	-	-
	GPT5	0.222	-	-	-
	RAG+DeepSeek-V3	0.239	-	-	-
	RAG+Qwen3-235B	0.598	-	-	-
	RAG+DeepSeek-R1	0.487			
	RAG+GPT5	0.422	-	-	-
	OPUS-PLLM-Llama3-8B	**0.880**	-	-	-
OPILoc	DeepSeek-V3	0.151	-	-	-
	DeepSeek-R1	0.281			
	Qwen3-235B	0.288	-	-	-
	GPT5	0.301	-	-	-
	RAG+DeepSeek-V3	0.304	-	-	-
	RAG+DeepSeek-R1	0.481			
	RAG+Qwen3-235B	0.584	-	-	-
	RAG+GPT5	0.510	-	-	-
	OPUS-PLLM-Llama3-8B	**0.883**	-	-	-
Swiss2024_Loc	DeepSeek-V3	0.079	-	-	-
	DeepSeek-R1	0.160			
	Qwen3-235B	0.162	-	-	-
	GPT5	0.105	-	-	-
	RAG+DeepSeek-V3	0.073	-	-	-
	RAG+DeepSeek-R1	0.260			
	RAG+Qwen3-235B	0.378	-	-	-
	RAG+GPT5	0.393	-	-	-
	OPUS-PLLM-Llama3-8B	**0.795**	-	-	-
		Gene Ontology (GO) Term			
CASPSimilar_GO	DeepSeek-V3		0.000	0.000	0.000
	DeepSeek-R1		0.000	0.000	0.000
	Qwen3-235B		0.000	0.000	0.000
	GPT5		0.000	0.000	0.000

	RAG+DeepSeek-V3	0.000	0.000	0.000
	RAG+DeepSeek-R1	0.034	0.035	0.031
	RAG+Qwen3-235B	0.024	0.024	0.024
	RAG+GPT5	0.012	0.009	0.010
	OPUS-PLLM-Llama3-8B	**0.846**	**0.863**	**0.848**
IDFilter_GO	DeepSeek-V3	0.001	0.000	0.001
	DeepSeek-R1	0.000	0.000	0.000
	Qwen3-235B	0.000	0.000	0.000
	GPT5	0.001	0.001	0.001
	RAG+DeepSeek-V3	0.007	0.007	0.007
	RAG+DeepSeek-R1	0.161	0.146	0.141
	RAG+Qwen3-235B	0.123	0.114	0.110
	RAG+GPT5	0.128	0.121	0.113
	OPUS-PLLM-Llama3-8B	**0.830**	**0.805**	**0.801**
UniProt_GO	DeepSeek-V3	0.000	0.000	0.000
	DeepSeek-R1	0.000	0.000	0.000
	Qwen3-235B	0.000	0.000	0.000
	GPT5	0.000	0.001	0.000
	RAG+DeepSeek-V3	0.019	0.018	0.017
	RAG+DeepSeek-R1	0.146	0.138	0.130
	RAG+Qwen3-235B	0.191	0.191	0.178
	RAG+GPT5	0.097	0.092	0.088
	OPUS-PLLM-Llama3-8B	**0.835**	**0.806**	**0.801**
Swiss2024_GO	DeepSeek-V3	0.001	0.001	0.001
	DeepSeek-R1	0.002	0.003	0.002
	Qwen3-235B	0.000	0.000	0.000
	GPT5	0.000	0.000	0.000
	RAG+DeepSeek-V3	0.044	0.049	0.044
	RAG+DeepSeek-R1	0.134	0.126	0.119
	RAG+Qwen3-235B	0.077	0.084	0.075
	RAG+GPT5	0.056	0.060	0.054
	OPUS-PLLM-Llama3-8B	**0.481**	**0.489**	**0.453**
UniProt Keyword				
CASPSimilar_Keyword	DeepSeek-V3	0.028	0.027	0.003
	DeepSeek-R1	0.002	0.003	0.002
	Qwen3-235B	0.047	0.073	0.046
	GPT5	0.037	0.021	0.026
	RAG+DeepSeek-V3	0.071	0.073	0.068
	RAG+DeepSeek-R1	0.227	0.176	0.183

	RAG+Qwen3-235B	0.136	0.101	0.109
	RAG+GPT5	0.110	0.092	0.094
	OPUS-PLLM-Llama3-8B	**0.884**	**0.859**	**0.862**
IDFilter_Keyword	DeepSeek-V3	0.032	0.030	0.028
	DeepSeek-R1	0.010	0.015	0.011
	Qwen3-235B	0.050	0.090	0.055
	GPT5	0.072	0.050	0.055
	RAG+DeepSeek-V3	0.131	0.244	0.163
	RAG+DeepSeek-R1	0.163	0.269	0.192
	RAG+Qwen3-235B	0.248	0.375	0.278
	RAG+GPT5	0.214	0.273	0.224
	OPUS-PLLM-Llama3-8B	**0.905**	**0.860**	**0.871**
UniProt_Keyword	DeepSeek-V3	0.046	0.068	0.045
	DeepSeek-R1	0.020	0.014	0.014
	Qwen3-235B	0.050	0.090	0.055
	GPT5	0.000	0.001	0.000
	RAG+DeepSeek-V3	0.177	0.252	0.197
	RAG+DeepSeek-R1	0.328	0.361	0.324
	RAG+Qwen3-235B	0.301	0.469	0.345
	RAG+GPT5	0.261	0.273	0.255
	OPUS-PLLM-Llama3-8B	**0.922**	**0.877**	**0.889**
Swiss2024_Keyword	DeepSeek-V3	0.041	0.065	0.041
	DeepSeek-R1	0.024	0.016	0.017
	Qwen3-235B	0.048	0.096	0.052
	GPT5	0.062	0.050	0.052
	RAG+DeepSeek-V3	0.186	0.297	0.216
	RAG+DeepSeek-R1	0.380	0.373	0.346
	RAG+Qwen3-235B	0.309	0.465	0.346
	RAG+GPT5	0.297	0.294	0.278
	OPUS-PLLM-Llama3-8B	**0.717**	**0.691**	**0.677**
Enzyme Commission (EC) Number				
EC_Price149	DeepSeek-V3	0.000	0.000	0.000
	DeepSeek-R1	0.000	0.000	0.000
	Qwen3-235B	0.000	0.000	0.000
	GPT5	0.000	0.000	0.000
	RAG+DeepSeek-V3	0.000	0.000	0.000
	RAG+DeepSeek-R1	0.020	0.020	0.020
	RAG+Qwen3-235B	0.040	0.040	0.040
	RAG+GPT5	0.027	0.027	0.027
	OPUS-PLLM-Llama3-	**0.262**	**0.262**	**0.262**

	8B			
EC_New392	DeepSeek-V3	0.000	0.000	0.000
	DeepSeek-R1	0.000	0.000	0.000
	Qwen3-235B	0.000	0.000	0.000
	GPT5	0.000	0.000	0.000
	RAG+DeepSeek-V3	0.009	0.009	0.009
	RAG+DeepSeek-R1	0.068	0.063	0.065
	RAG+Qwen3-235B	0.039	0.036	0.037
	RAG+GPT5	0.066	0.061	0.063
	OPUS-PLLM-Llama3-8B	**0.495**	**0.479**	**0.482**

Table S12. Performance comparison between OPUS-PLLM and leading general-purpose large language models (LLMs) with and without retrieval-augmented generation (RAG) on functional description generation.

Benchmarks	Models	R1	R2	RL	RLS	B	BP	BR	BF	M
Functional Description										
CASPSimilar_Description	DeepSeek-V3	0.081	0.005	0.067	0.067	0.000	0.727	0.719	0.722	0.067
	DeepSeek-R1	0.050	0.003	0.044	0.045	0.000	0.715	0.690	0.701	0.036
	Qwen3-235B	0.081	0.007	0.065	0.065	0.000	0.722	0.711	0.716	0.058
	GPT5	0.063	0.005	0.046	0.047	0.000	0.717	0.717	0.716	0.054
	RAG+DeepSeek-V3	0.134	0.014	0.100	0.100	0.007	0.700	0.728	0.713	0.118
	RAG+DeepSeek-R1	0.147	0.019	0.105	0.109	0.009	0.701	0.735	0.717	0.131
	RAG+Qwen3-235B	0.210	0.040	0.151	0.149	0.020	0.737	0.757	0.746	0.182
	RAG+GPT5	0.187	0.033	0.135	0.135	0.012	0.728	0.747	0.737	0.135
	OPUS-PLLM-Llama3-8B	**0.832**	**0.795**	**0.817**	**0.816**	**0.618**	**0.947**	**0.948**	**0.947**	**0.816**
IDFilter_Description	DeepSeek-V3	0.110	0.011	0.083	0.083	0.002	0.732	0.701	0.715	0.101
	DeepSeek-R1	0.074	0.010	0.062	0.062	0.001	0.724	0.682	0.701	0.065
	Qwen3-235B	0.107	0.013	0.083	0.083	0.006	0.724	0.694	0.708	0.100
	GPT5	0.103	0.013	0.081	0.081	0.004	0.720	0.707	0.713	0.109
	RAG+DeepSeek-V3	0.200	0.095	0.179	0.179	0.082	0.741	0.739	0.738	0.160
	RAG+DeepSeek-R1	0.214	0.101	0.183	0.189	0.079	0.741	0.753	0.746	0.194
	RAG+Qwen3-235B	0.204	0.097	0.184	0.183	0.056	0.716	0.721	0.717	0.158
	RAG+GPT5	0.254	0.108	0.202	0.202	0.057	0.785	0.783	0.783	0.206
	OPUS-PLLM-Llama3-8B	**0.766**	**0.729**	**0.756**	**0.756**	**0.596**	**0.928**	**0.925**	**0.926**	**0.752**
UniProt_Description	DeepSeek-V3	0.113	0.011	0.085	0.086	0.003	0.745	0.701	0.721	0.100
	DeepSeek-R1	0.060	0.010	0.051	0.052	0.001	0.740	0.679	0.707	0.048
	Qwen3-235B	0.104	0.014	0.084	0.084	0.003	0.740	0.696	0.716	0.086
	GPT5	0.092	0.011	0.072	0.072	0.003	0.734	0.710	0.721	0.094
	RAG+DeepSeek-V3	0.174	0.066	0.139	0.139	0.038	0.714	0.724	0.718	0.202
	RAG+DeepSeek-R1	0.195	0.079	0.153	0.156	0.039	0.724	0.740	0.731	0.221
	RAG+Qwen3-235B	0.202	0.096	0.167	0.167	0.059	0.728	0.737	0.732	0.227
	RAG+GPT5	0.259	0.103	0.197	0.197	0.061	0.778	0.782	0.779	0.234
	OPUS-PLLM-Llama3-8B	**0.796**	**0.760**	**0.785**	**0.785**	**0.656**	**0.935**	**0.935**	**0.934**	**0.784**
Swiss2024_Description	DeepSeek-V3	0.122	0.012	0.085	0.085	0.003	0.729	0.680	0.703	0.097
	DeepSeek-R1	0.056	0.009	0.047	0.048	0.000	0.737	0.657	0.693	0.039
	Qwen3-235B	0.100	0.013	0.079	0.079	0.001	0.740	0.672	0.704	0.067
	GPT5	0.090	0.009	0.068	0.068	0.001	0.739	0.686	0.711	0.075
	RAG+DeepSeek-V3	0.173	0.035	0.123	0.124	0.020	0.713	0.703	0.707	0.174
	RAG+DeepSeek-R1	0.197	0.060	0.145	0.148	0.046	0.738	0.719	0.728	0.177

RAG+Qwen3-235B	0.174	0.039	0.126	0.126	0.035	0.718	0.707	0.712	0.175
RAG+GPT5	0.162	0.037	0.117	0.117	0.017	0.758	0.717	0.736	0.123
OPUS-PLLM-Llama3-8B	**0.273**	**0.157**	**0.236**	**0.235**	**0.090**	**0.781**	**0.751**	**0.764**	**0.210**

Table S13. Comparison of OPUS-PLLM against several leading discriminative models.

Benchmarks	Models	Accuracy	Precision	Recall	F1-Score
		Subcellular Localization			
	LocPro	0.877	-	-	-
	DeepLoc2.0	0.857	-	-	-
DeepLoc	DeepLoc2.1	0.857	-	-	-
	OPUS-PLLM-Gala6.7B	0.874	-	-	-
	OPUS-PLLM-Llama3-8B	**0.880**	-	-	
	LocPro	0.873	-	-	-
	DeepLoc2.0	0.859	-	-	-
OPILoc	DeepLoc2.1	0.861	-	-	-
	OPUS-PLLM-Gala6.7B	0.869	-	-	-
	OPUS-PLLM-Llama3-8B	**0.883**	-	-	-
	LocPro	0.748	-	-	-
	DeepLoc2.0	0.766	-	-	-
Swiss2024_Loc	DeepLoc2.1	0.761	-	-	-
	OPUS-PLLM-Gala6.7B	**0.812**	-	-	-
	OPUS-PLLM-Llama3-8B	0.795	-	-	-
		Gene Ontology (GO) Term			
	DeepGO-SE		0.206	0.310	0.232
CASPSimilar_GO	OPUS-PLLM-Gala6.7B		0.832	0.863	0.838
	OPUS-PLLM-Llama3-8B		**0.846**	**0.863**	**0.848**
	DeepGO-SE		0.420	0.532	0.426
IDFilter_GO	OPUS-PLLM-Gala6.7B		0.791	0.799	0.777
	OPUS-PLLM-Llama3-8B		**0.830**	**0.805**	**0.801**
	DeepGO-SE		0.416	0.506	0.414
UniProt_GO	OPUS-PLLM-Gala6.7B		0.828	0.806	0.799
	OPUS-PLLM-Llama3-8B		**0.835**	**0.806**	**0.801**
	DeepGO-SE		0.342	0.526	0.367
Swiss2024_GO	OPUS-PLLM-Gala6.7B		0.472	0.489	0.445
	OPUS-PLLM-Llama3-8B		**0.481**	**0.489**	**0.453**
		Enzyme Commission (EC) Number			
	GraphEC		0.168	0.201	0.178
	DeepECtransformer		0.305	0.305	0.305
	CLEAN		**0.531**	**0.434**	**0.452**
EC_Price149	OPUS-GO		0.345	0.413	0.365
	OPUS-PLLM-Gala6.7B		0.349	0.346	0.347
	OPUS-PLLM-Llama3-8B		0.262	0.262	0.262
	GraphEC		0.326	0.394	0.339
EC_New392	DeepECtransformer		0.400	0.382	0.386
	CLEAN		**0.561**	0.509	0.504

OPUS-GO	0.504	**0.540**	0.511
OPUS-PLLM-Gala6.7B	0.545	0.533	**0.536**
OPUS-PLLM-Llama3-8B	0.495	0.479	0.482

Table S14. Performance comparison between OPUS-PLLM and DeepGO-SE on the DeepGO-SE test set. OPUS-PLLM is trained using the same data as DeepGO-SE (replacing the GO term data in OPUS-InstructionCorpus), with the BP, MF, and CC datasets combined into a unified dataset.

Benchmarks	Models	Precision	Recall	F1-Score
Gene Ontology (GO) Term				
DeepGO-SE	DeepGO-SE	**0.457**	0.391	0.367
(Test set)	OPUS-PLLM-Llama3-8B	0.395	**0.495**	**0.406**

Table S15. Comparison of mean Matthews Correlation Coefficient (MCC) and low-frequency-term MCC (frequency < 0.02) for generative (OPUS-PLLM-Llama3-8B) and discriminative models utilizing ESM2 representations.

Benchmarks	Models	MCC (mean)	MCC (low-frequency-term)
CASPSimilar_GO	ESM2	0.258	0.050
	OPUS-PLLM-Llama3-8B	**0.347**	**0.055**
IDFilter_GO	ESM2	0.372	0.361
	OPUS-PLLM-Llama3-8B	**0.444**	**0.434**
UniProt_GO	ESM2	0.341	0.339
	OPUS-PLLM-Llama3-8B	**0.391**	**0.389**
Swiss2024_GO	ESM2	0.186	0.182
	OPUS-PLLM-Llama3-8B	**0.245**	**0.240**
CASPSimilar_Keyword	ESM2	0.500	0.188
	OPUS-PLLM-Llama3-8B	**0.666**	**0.330**
IDFilter_Keyword	ESM2	0.694	0.658
	OPUS-PLLM-Llama3-8B	**0.780**	**0.759**
UniProt_Keyword	ESM2	0.679	0.659
	OPUS-PLLM-Llama3-8B	**0.748**	**0.732**
Swiss2024_Keyword	ESM2	0.487	0.460
	OPUS-PLLM-Llama3-8B	**0.519**	**0.492**
EC_Price149	ESM2	0.221	0.116
	OPUS-PLLM-Llama3-8B	**0.314**	**0.281**
EC_New392	ESM2	0.436	0.446
	OPUS-PLLM-Llama3-8B	**0.452**	**0.469**

Table S16. Summary of datasets used for benchmarking OPUS-PLLM.

Benchmarks	Sample counts	Number of labels	Metrics
Subcellular Localization			
DeepLoc	2442	10	Accuracy
OPI_Loc	2695		
Swiss2024_Loc	904		
Gene Ontology (GO) Term			
CASPSimilar_GO	130	28837	Precision, Recall, F1-score
IDFilter_GO	922		
UniProt_GO	3515		
Swiss2024_GO	1607		
UniProt Keyword			
CASPSimilar_Keyword	130	1176	Precision, Recall, F1-score
IDFilter_Keyword	922		
UniProt_Keyword	3515		
Swiss2024_Keyword	1607		
Enzyme Commission (EC) Number			
EC_Price149	149	5204	Precision, Recall, F1-score
EC_New392	392		
Functional Description			
CASPSimilar_Description	130	N/A	ROUGE-1, ROUGE-2, ROUGE-L, ROUGE-Lsum, BLEU, BERTScore -Precision, BERTScore-Recall, BERTScore-F1, METEOR
IDFilter_Description	922		
UniProt_Description	3515		
Swiss2024_Descripstion	1607		
Multiple-choice question answering (MCQA)			
Swiss2024-MCQA	1684	N/A	Accuracy

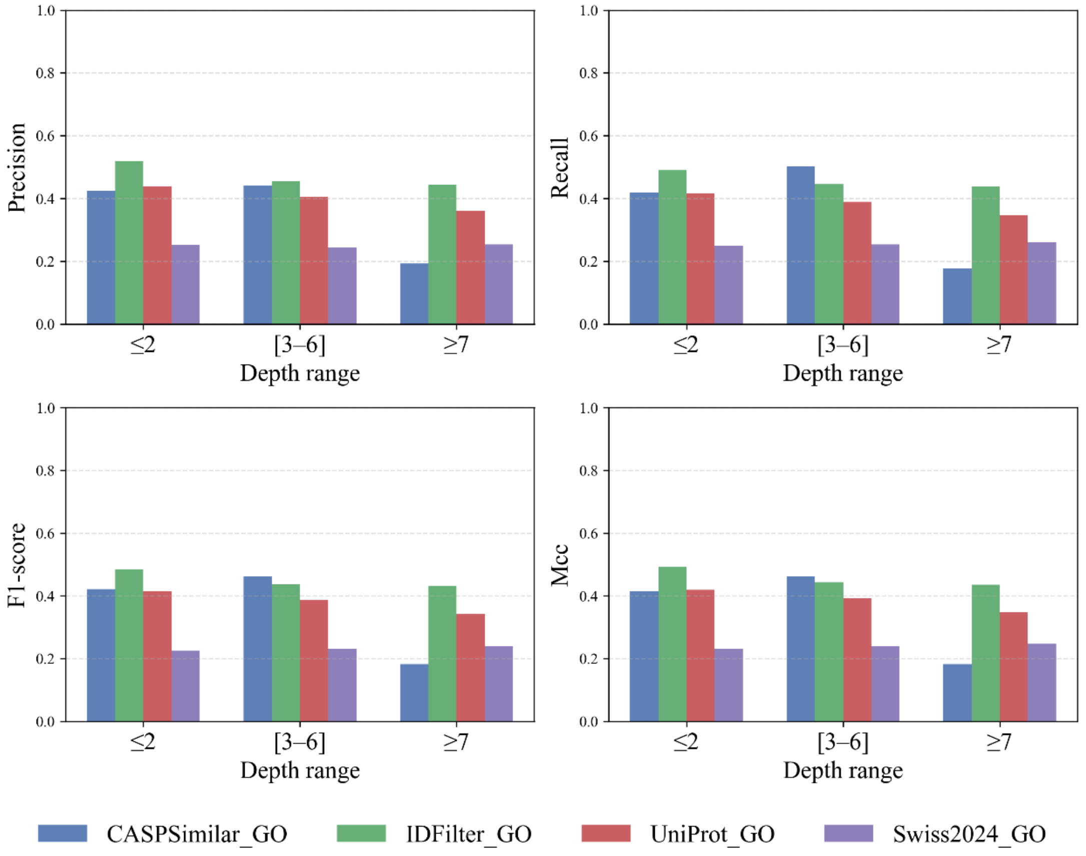

Fig S1. Performance of OPUS-PLLM across different GO term depths. Based on the hierarchical depth of GO terms within the ontology, labels are categorized into three classes: shallow (depth ≤ 2), intermediate (3 ≤ depth ≤ 6), and deep (depth ≥ 7). Weighted averages of Precision, Recall, F1-score, and MCC are calculated for each depth range across the four datasets.
