# Supplementary material for: Advancing generative large language models toward discriminative performance in protein function prediction
Source: Genome Biol. 2026 May 21;27:226. doi: 10.1186/s13059-026-04109-8 (PMC13366677; doi:10.1186/s13059-026-04109-8)
Supplement: Supplementary file 2 — Additional file 2. Detailed descriptions of evaluation metrics. [file 13059_2026_4109_MOESM2_ESM.pdf]

Additional file 2:

Advancing Generative Large Language Models Toward Discriminative Performance in Protein Function Prediction

Lv *et al*.

Detailed descriptions of evaluation metrics

In this study, while our model is a generative large language model (LLM), the tasks under investigation include both classification and text generation. We therefore adopt metrics commonly used for classification tasks, complemented by standard evaluation metrics for LLMs. First, we define general notation:

The test set is denoted as $\mathcal{D} = \{(x_i, y_i)\}_{i=1}^{m}$, where m represents the number of samples. For single-label classification (subcellular localization prediction), let K denote the number of classes. The true label of sample of sample i is $y_i \in \{1, \dots, K\}$, and the predicted label is $\hat{y}_i \in \{1, \dots, K\}$. For multi-label classification (GO term prediction, EC number prediction, UniProt keyword prediction), let L denote the total number of unique labels. Each sample is represented by binary vectors: $\mathbf{y}_i = (y_{i1}, \dots, y_{iL}) \in \{0,1\}^L$ (true label) and $\hat{\mathbf{y}}_i = (\hat{y}_{i1}, \dots, \hat{y}_{iL}) \in \{0,1\}^L$ (predicted label), where $y_{ij} = 1$ indicates sample i is annotated with label j. For functional description generation, the reference text for sample i is r_i, and the model-generated text is c_i. Any text-based metric is denoted as $s(r_i, c_i)$.

For subcellular localization prediction, we use *Accuracy* to quantify overall prediction performance:

$$\text{Accuracy} = \frac{1}{m}\sum_{i=1}^{m} \mathbb{I}\left(\hat{y}_i = y_i\right)$$

where $\mathbb{I}(\cdot)$ denotes the indicator function (returning 1 if the condition is satisfied, 0 otherwise).

For GO term prediction, EC number prediction, and UniProt keyword prediction, we adopt Precision (P), Recall (R), and F1-score (F1) for per-sample evaluation, then average across the test set to obtain corpus-level results. For a single sample:

Define true positives (TP), false positives (FP), and false negatives (FN):

$$TP = \sum_{j=1}^{L} \mathbb{I}\left(y_j = 1 \land \hat{y}_j = 1\right)$$

$$FP = \sum_{j=1}^{L} \mathbb{I}\left(y_j = 0 \land \hat{y}_j = 1\right)$$

$$FN = \sum_{j=1}^{L} \mathbb{I}\,(y_j = 1 \wedge \hat{y}_j = 0)$$

Then the Precision (P), Recall (R), and F1-score (F1) for this sample are defined as:

$$P = \frac{TP}{TP + FP}$$

$$R = \frac{TP}{TP + FN}$$

$$F1 = \frac{2PR}{P + R}$$

For functional description generation, we evaluate generated text quality using 9 widely adopted LLM metrics. For each sample, we compute $s(r_i, c_i)$, and the corpus-level score is the average across all test samples is $S = \frac{1}{m}\sum_i s\,(r_i, c_i)$.

1) ROUGE series: ROUGE-1 (R1, unigram recall), ROUGE-2 (R2, bigram overlap), ROUGE-L (RL, sentence-level longest common subsequence), and ROUGE-Lsum (RLS, document-level longest common subsequence across concatenated references).

For ROUGE-1 and ROUGE-2, let $\mathcal{G}_n(t)$ denote the multiset of n-grams (including repetitions) in text t, and $\mathrm{cnt}_t(g)$ denote the count of n-gram g in t. The sample-level score is:

$$\mathrm{ROUGE} - n(r, c) = \frac{\sum_{g \in \mathcal{G}_n(r)} \min(\,\mathrm{cnt}_r(g), \mathrm{cnt}_c(g))}{\sum_{g \in \mathcal{G}_n(r)} \mathrm{cnt}_r(g)}$$

Corpus-level score (average of sample-level scores):

$$\mathrm{R}n_{corpus} = \frac{1}{N}\sum_{k=1}^{N} \mathrm{ROUGE} - n\left(r^{(k)}, c^{(k)}\right)$$

For ROUGE-L, let $\mathrm{LCS}(r, c)$ denote the length of the LCS between r and c, and |r| denote the reference length (tokens). Sample-level score:

$$\mathrm{ROUGE} - L(r, c) = \frac{\mathrm{LCS}(r, c)}{|r|}$$

Corpus-level score:

$$\mathrm{RL}_{corpus} = \frac{1}{N}\sum_{k=1}^{N} \mathrm{ROUGE} - L\left(r^{(k)}, c^{(k)}\right)$$

For ROUGE-Lsum, concatenating all references for a sample into $\tilde{r}$, and denote

generated text as $\tilde{c}$. Sample-level score:

$$\text{ROUGE} - \text{Lsum}(\tilde{r}, \tilde{c}) = \frac{\text{LCS}(\tilde{r}, \tilde{c})}{|\tilde{r}|}$$

Corpus-level score:

$$\text{RLS}_{corpus} = \frac{1}{N}\sum_{k=1}^{N} \text{ROUGE} - \text{Lsum}\left(\tilde{r}^{(k)}, \tilde{c}^{(k)}\right)$$

2) BLEU (B) measures n-gram precision with a brevity penalty (BP) to address over-short generated texts. We use BLEU-4 (max $\text{n} - \text{gram} = 4$, equal weights $\text{w}_n = 1/4$).

Modified n-gram precision:

$$\text{mp}_n(r, c) = \frac{\sum_{g \in \mathcal{G}_n(c)} \min(\text{cnt}_\text{c}(g), \text{cnt}_\text{r}(g))}{\sum_{g \in \mathcal{G}_n(c)} \text{cnt}_\text{c}(g)}$$

BLEU score:

$$\text{BLEU}(r, c) = \text{BP} \cdot \exp\left(\sum_{n=1}^{4} w_n \log(\text{mp}_n\,(r, c))\right)$$

Brevity penalty:

$$\text{BP} = \begin{cases} 1, & if\ |c| > |r|, \\ \exp\left(1 - \frac{|r|}{|c|}\right), & if\ |c| \leq |r|. \end{cases}$$

Corpus-level score (concatenate all texts first):

$$\text{B}_{corpus} = \text{BLEU}\left(\text{concat}\left(r^{(k)}\right), \text{concat}\left(c^{(k)}\right)\right)$$

3) BERTScore series quantify semantic similarity using biomedical domain-specific embeddings (BioBERT-large-cased-v1 [48]). We use uniform token weights (IDF weights are ineffective for short domain texts):

Sample-level metrics (Precision: BP, Recall: BR, F1: BF):

$$\text{BP}(r, c) = \frac{\sum_{t=1}^{|c|} w_t \max_{1 \leq s \leq |r|} \cos(u_t, v_s)}{\sum_{t=1}^{|c|} 1}$$

$$\text{BR}(r, c) = \frac{\sum_{s=1}^{|r|} \widetilde{w}_s \max_{1 \leq t \leq |c|} \cos(u_t, v_s)}{\sum_{s=1}^{|r|} 1}$$

$$\mathrm{BF}(r,c)=\frac{2\cdot\mathrm{BP}(r,c)\cdot\mathrm{BR}(r,c)}{\mathrm{BP}(r,c)+\mathrm{BR}(r,c)}$$

where u_t and v_s are token embeddings of c and r, respectively.

Corpus-level scores (average of sample-level scores):

$$\mathrm{BP}_{corpus}=\frac{1}{N}\sum_{k=1}^{N}\mathrm{BP}\left(r^{(k)},c^{(k)}\right)$$

$$\mathrm{BR}_{corpus}=\frac{1}{N}\sum_{k=1}^{N}\mathrm{BR}\left(r^{(k)},c^{(k)}\right)$$

$$\mathrm{BF}_{corpus}=\frac{1}{N}\sum_{k=1}^{N}\mathrm{BF}\left(r^{(k)},c^{(k)}\right)$$

4) METEOR (M) integrates exact/stem/synonym matching and a fragmentation penalty to balance lexical accuracy and coherence.

Unigram precision/recall:

$$P(r,c)=\frac{m}{|c|},R(r,c)=\frac{m}{|r|}$$

where m is the number of matched unigrams.

Weighted harmonic mean:

$$F_{mean}(r,c)=\frac{10\,P(r,c)\,R(r,c)}{R(r,c)+9\,P(r,c)}$$

Let ch be the number of contiguous matched chunks. The penalty term is:

$$\mathrm{Pen}(r,c)=\gamma\left(\frac{ch}{m}\right)^{\beta}$$

Sample-level score:

$$\mathrm{METEOR}(r,c)=(1-\mathrm{Pen}(r,c))\cdot F_{mean}(r,c)$$

Corpus-level score:

$$\mathrm{M}_{corpus}=\frac{1}{N}\sum_{k=1}^{N}\mathrm{METEOR}\left(r^{(k)},c^{(k)}\right)$$

For the subcellular localization task, which is a single-label, 10-class classification problem where each protein is assigned to exactly one location, accuracy is a direct and comprehensive metric. The accuracy measures the exact match between the prediction and the single ground-truth label, effectively capturing the model's overall performance in this closed-set scenario.

In contrast, the tasks of GO term, UniProt keyword, and EC number prediction are inherently multi-label. A single protein can be associated with multiple correct functional annotations, and the label distribution is highly sparse. In such settings, accuracy becomes an inadequate and often misleading metric. Its binary, all-or-nothing nature fails to account for partial correctness and is heavily influenced by threshold choices and extreme class imbalance. Therefore, for these tasks, we adhere to the established standards in the field by employing precision, recall, and F1-score, that are specifically designed to robustly evaluate performance in multi-label, imbalanced classification problems.
